# Platinum(IV) Derivatives of [Pt(1*S*,2*S*-diaminocyclohexane)(5,6-dimethyl-1,10-phenanthroline)] with Diclofenac Ligands in the Axial Positions: A New Class of Potent Multi-action Agents Exhibiting Selectivity to Cancer Cells

**DOI:** 10.1021/acs.jmedchem.3c00269

**Published:** 2023-06-07

**Authors:** Hana Kostrhunova, Brondwyn S. McGhie, Lenka Markova, Olga Novakova, Jana Kasparkova, Janice R. Aldrich-Wright, Viktor Brabec

**Affiliations:** † Institute of Biophysics, Czech Academy of Sciences, Kralovopolska 135, CZ-61200 Brno, Czech Republic; ‡ School of Science, Western Sydney University, Penrith South DC 1797, New South Wales, Australia; § Department of Biophysics, Faculty of Science, Palacky University, Slechtitelu 27, 783 71 Olomouc, Czech Republic

## Abstract

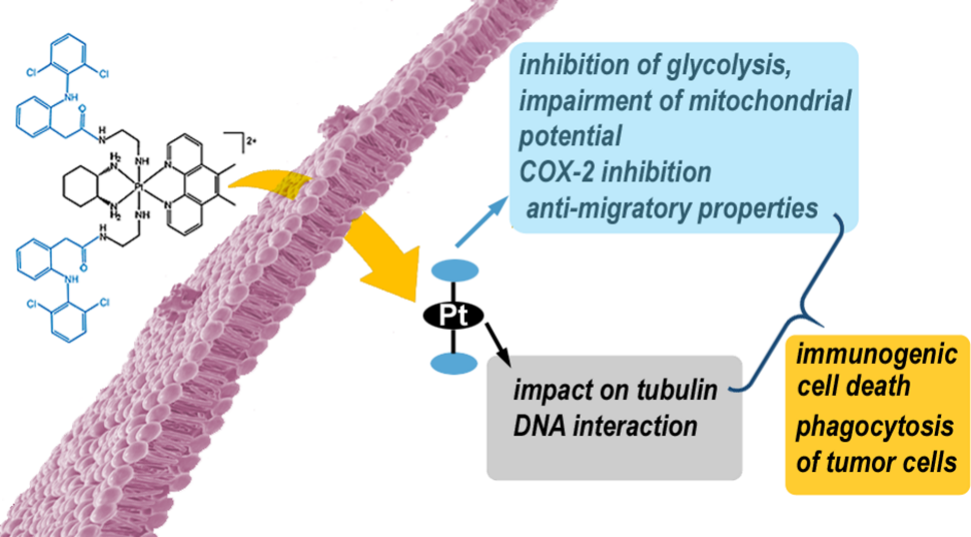

The platinum(II) complex [Pt(1*S*,2S-diaminocyclohexane)(5,6-dimethyl-1,10-phenanthroline)]^2+^ (Pt^II^56MeSS, **1**) exhibits high potency across numerous cancer cell lines acting by a multimodal mechanism. However, **1** also displays side toxicity and in vivo activity; all details of its mechanism of action are not entirely clear. Here, we describe the synthesis and biological properties of new platinum(IV) prodrugs that combine **1** with one or two axially coordinated molecules of diclofenac (DCF), a non-steroidal anti-inflammatory cancer-selective drug. The results suggest that these Pt(IV) complexes exhibit mechanisms of action typical for Pt(II) complex **1** and DCF, simultaneously. The presence of DCF ligand(s) in the Pt(IV) complexes promotes the antiproliferative activity and selectivity of **1** by inhibiting lactate transporters, resulting in blockage of the glycolytic process and impairment of mitochondrial potential. Additionally, the investigated Pt(IV) complexes selectively induce cell death in cancer cells, and the Pt(IV) complexes containing DCF ligands induce hallmarks of immunogenic cell death in cancer cells.

## Introduction

Platinum(II) anticancer drugs like cisplatin, carboplatin, and oxaliplatin are among the most widely used antitumor chemotherapeutics; approximately half of all chemotherapeutic treatment exploits a platinum drug.^1^ However, a number of attendant disadvantages exist that limit the clinical application of platinum(II)-based drugs. Among them, toxic adverse side effects, inherent and acquired resistance, narrow spectrum of activity, ineffectiveness toward cancer stem cells (CSCs), and lack of antimetastatic activity represent the most limiting problems. To overcome these limitations, many platinum complexes have been prepared and tested for anticancer activity and their mechanism of action.

The current clinically used platinum(II) anticancer drugs, cisplatin, carboplatin, and oxaliplatin, elicit their activity through inhibition of DNA replication and transcription by the formation of coordinate bonds between the drug and DNA. With the aim to design new compounds demonstrating distinct mechanisms of action and circumventing the limitations, different molecular strategies to inhibit cellular proliferation, such as intercalation, are currently under investigation.

**Figure 1:**
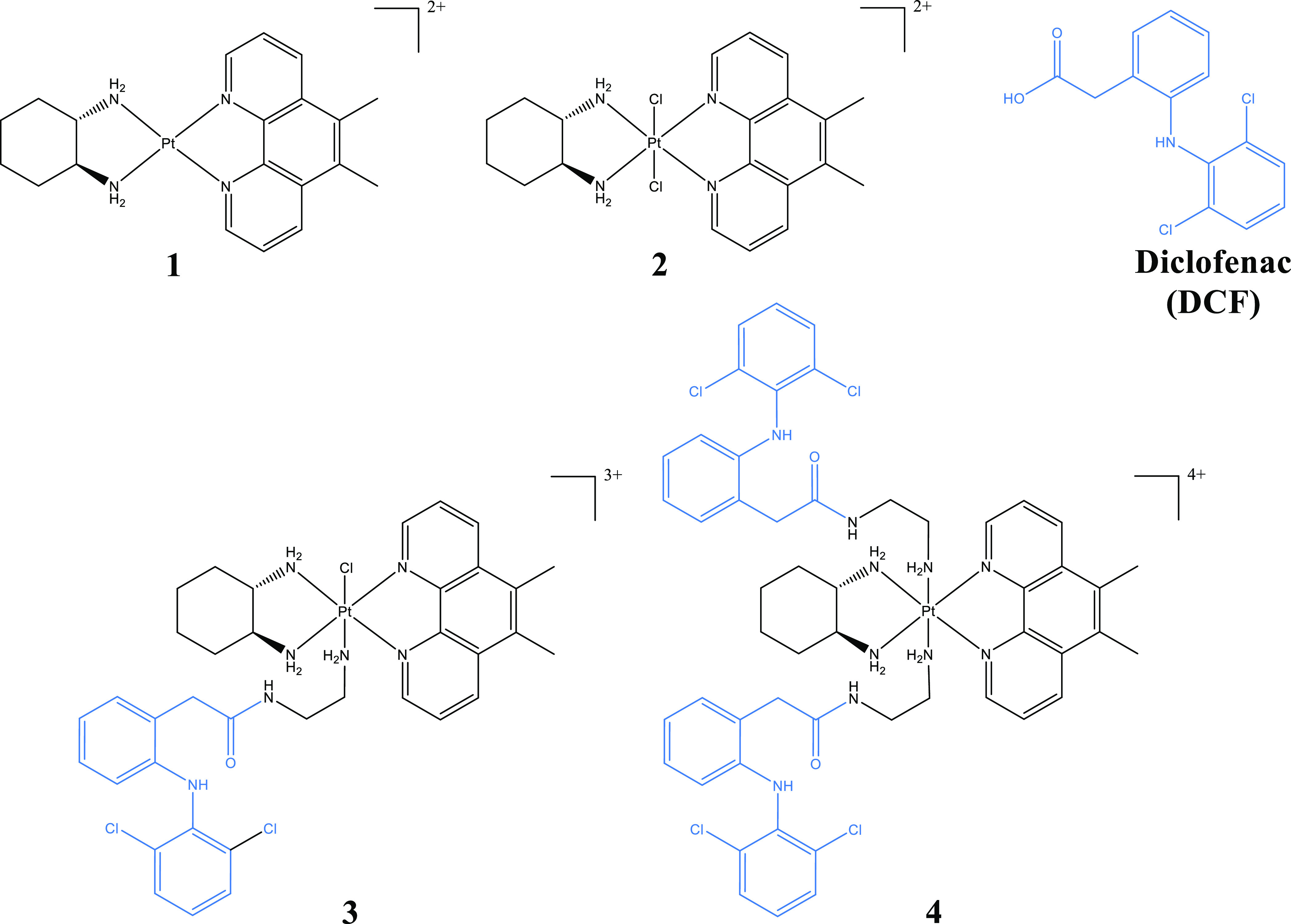
Structures of compounds investigated in this work. The counterions (Cl^–^) have been omitted for clarity.

Extensive studies on unconventional platinum(II) complexes of general formula [Pt(P_L_)(A_L_)]^2+^, where P_L_ is a polyaromatic ligand, and A_L_ is an ancillary ligand, have yielded some promising results. A group of complexes combining a phenanthroline-based ligand, such as 5,6-dimethyl-1,10-phenanthroline (56Me_2_Phen) (P_L_), and a 1,2-diaminocycloalkane (DACH) ligand, such as 1*S*,2*S*-diaminocyclohexane (SS-DACH) (A_L_), showed impressive activities against human tumor cell lines.^2,3^ The lead complex [Pt(1*S*,2S-diaminocyclohexane)(5,6-dimethyl-1,10-phenanthroline)]^2+^ (Pt^II^56MeSS, complex **1**; Figure 1) has been shown to act by a multimodal mechanism involving the impact on mitochondrial and cell cycle proteins,^4^ cytoskeleton impairment,^4,5^ disruption of iron and copper metabolism along with suppression of sulfur-containing amino acids,^6^ and also interaction with nuclear DNA.^7,8^ Moreover, the upregulation of fatty acyl-CoA synthetase FACL4 by Pt(II) complex **1** was recently described.^9^

Although Pt(II) complex **1** exhibits high potency across numerous cancer cell lines, its in vivo activity is not entirely clear. For instance, administration of Pt(II) complex **1** revealed no antitumor activity in BD-IX rats with peritoneal carcinomatosis,^10^ while a potent anticancer effect on an oral cancer xenograft model on BALB/c nude mice was reported.^9^ Importantly, the in vivo activity of Pt(II) complex **1** was significantly improved by its oxidation to the platinum(IV) analogue.^11^

Generally, platinum(IV) complexes exhibit favorable chemical properties compared to Pt(II) analogues. Their kinetic inertness prevents their inactivation by extracellular off-target molecules that reduce undesired side effects.^12,13^ Importantly, the two additional coordination sites provide the potential for the conjugation of additional ligands. These ligands may significantly advance the resulting anticancer activity via improving chemical properties (lipophilicity, reduction kinetics)^14−17^ and/or due to their own biological activity yielding dual or multi-action agents. Several multi-action Pt(IV) complexes have already been reported.^18−21^ Pt(IV) prodrugs are reduced by an intracellular reducing environment to generate Pt(II) drugs, and simultaneously the coordinated bioactive ligands are released. This results in a combined effect unachievable by administration of the mixture of two or more drugs, as drugs administered as a mixture of single agents may not necessarily reach the targeted sites simultaneously in appropriate dose and ratio.

It is generally accepted that inflammatory cells and cellular mediators of inflammation are prominent constituents of the microenvironment of all tumors.^22^ Therefore, anti-inflammatory agents, including non-steroidal anti-inflammatory drugs (NSAIDs), are coming into focus when designing new chemotherapy strategies.^23^ NSAIDs have shown promise in cancer prevention, but there is now emerging evidence that such drugs may be useful in actually treating cancer. Although the main anti-inflammatory mechanism of action of NSAIDs is the inhibition of cyclooxygenases (COX-1 and COX-2 isoenzymes), they likely execute their anticancer activity via both COX-dependent and COX-independent mechanisms. The potential COX-2-independent mechanism of NSAIDs’ antineoplastic action includes the downregulation of proto-oncogenes and transcriptional factors such as PPARδ, NF-κB, PAR-4, and *Bcl-2*.^24^

Several Pt(II) or Pt(IV) complexes combining a Pt moiety with some NSAIDs (aspirin, indomethacin, ibuprofen, etc.) have also been designed and tested.^25−30^ Diclofenac, sodium {2-[(2,6-dichlorophenyl)amino]phenyl}-acetate (DCF, Figure 1) is an NSAID frequently used to treat pain; it is cost-effective and available as a generic drug.^31^ In addition to the antitumor effect attributed to the inhibition of COX, DCF has also shown novel COX-independent effects caused by its influencing of glucose metabolism, particularly due to lactate transporter inhibition.^31,32^ Interestingly, so far, no other NSAIDs have been shown to affect glucose metabolism. Moreover, there is considerable evidence that DCF binds COX-2 via a different mechanism to other NSAIDs;^33^ therefore, DCF-specific anticancer mechanisms of action, including anti-angiogenic and pro-apoptotic action, inhibition of Myc expression, immunomodulation activity,^31^ and inhibition of microtubule polymerization,^34^ are frequently discussed. Given such multiple mechanisms, particularly with respect to its effect on angiogenesis and the immune system, DCF can be considered a drug with a huge potential to treat cancer.^35^ For these reasons, the conjugation of DCF with a Pt(IV)-based anticancer drug also appears to be advantageous. Recently, platinum(II) complexes comprising DCF have been described;^36^ the complex with DCF molecules conjugated to platinum through the carboxylic group exhibited elevated cytotoxicity as well as selectivity toward cancer cells, as compared to clinically used cisplatin. Also, interestingly, a series of Pt(IV) prodrugs derived from cisplatin with NSAIDs, naproxen, DCF, and flurbiprofen, in the axial position were shown to exhibit superior antiproliferative activity compared to parental cisplatin as well as an ability to overcome tumor cell line resistance to cisplatin.^37^

Here, we describe the synthesis and biological properties of new prodrugs that combine two bioactive constituents: [Pt(1*S*,2*S*-diaminocyclohexane)(5,6-dimethyl-1,10-phenanthroline)(X)(Y)]^2+^ (Pt^Iv^56MeSS) with one or two axially coordinated DCFs (Figure 1). These complexes were designed with the intention of enriching and improving the biological action of Pt(II) complex **1**. As Pt(II) complex **1** displays some side toxicity,^10^ it is reasonable to assume that oxidation and conjugation with cancer-selective DCF could potentially improve its preference for cancerous over non-cancerous cells. In addition, conjugated DCF could bring additional added value related to its intrinsic biological action.

## Results and Discussion

### Synthesis and Characterization of Diclofenac Amide (enDCF)

Attempts to conjugate DCF directly to the Pt center resulted in a Pt complex that was unstable, so we used an ethylenediamine linker to conjugate bulky ligands to the Pt center. DCF was dissolved in a minimal amount of chloroform before 2 equiv of 1,2-ethylenediamine (en) were added at room temperature. The reaction occurs instantaneously, giving an excellent yield. The solution, left overnight, produced large colorless crystals, which were filtered and washed with chloroform. Some crystals were slightly beige, so these were recrystallized in ethanol to produce colorless crystals with a 95% yield. To confirm that enDCF had formed, proton NMR spectra were measured (Figures S1 and S2). The proton resonances in the ^1^H spectra for enDCF are shifted downfield when compared to that of DCF, as shown in Figure S3. The H13 and H4 peaks are significantly merged for enDCF, whereas in the DCF spectra, the individual peaks are discernible. This confirmed the purity of enDCF for further synthesis.

### Synthesis of Pt(IV) Complexes

The intermediate, Pt^IV^56MeSSCl_2_ (complex **2**; Figure 1), was isolated utilizing previously published methods, using *N*-chloro succinimide to oxidize Pt(II) complex **1**.^38^ Further purification was not required because, upon precipitation, the succinimide by-product was separated from the dichloride intermediate, and the resulting solution was instead dried under vacuum. Dimethyl sulfoxide (DMSO) was added to the dried Pt(IV) complex **2**.

HPLC and NMR characterizations were consistent with what was expected and are provided in Figures S3–S8. Thus, all compounds were >95% pure by NMR and HPLC analyses. NMR reduction studies were also undertaken where excess equivalents of reducing agents GSH or ascorbic acid were added to the sample before undertaking multiple NMR measurements over a 48 h period at 37 °C. These reducing agents were chosen to mimic the reducing capacity of the extracellular environment of the blood and tissue. The resulting spectra showed that even upon the addition of up to 10 equiv of the reducing agent to Pt^IV^56MeSS(DCF)_2_ (complex **4**, Figure 1), the compound had not been reduced (Supporting Information, Figures S9–S16). This indicates that the complex is stable at biologically relevant temperatures and in the presence of the reducing agents. However, once inside the cell, reactions with intracellular components might be induced, rendering the prodrug biodegradable. This strongly indicates that the Pt(IV) prodrug is stable in extracellular environments, but free DCF, when the Pt(IV) prodrug enters the cell, can be cleaved off. Therefore, the complex is unlikely to be reduced in the bloodstream. Monitoring the Pt(IV) peak, falling as the Pt(II) peak rises, would be ideal for further proof of the stability of these complexes, but the Pt(IV) resonance falls outside the detectable range of our instrument. After searching the entire scannable range for a Pt(IV) resonance, one could not be found; however, each sample produced a Pt(II) resonance at −2820 ppm even after 2.5–3 weeks (Supporting Information, Figure S1), demonstrating, indirectly, that the platinum(IV) complex can be reduced over time (3 weeks) in extracellular environments (Supporting Information, Figure S1).

To monitor the fate of the Pt(IV) complexes inside the cells, the effect of incubating the Pt(IV) complex **4** with HeLa cell extract was followed by HPLC. Since the high-molecular fraction of the cell extract plays a major role in the reduction of Pt(IV) complexes (Wexselblatt, E.; Gibson, D. *J. Inorg. Biochem.***2012,***117,* 220–229), we incubated complex **4** with this fraction (MW > 3 kDa). The results suggest that in the cellular environment, DCF is likely released from **4** and further metabolized to form the same products as DCF (Figure S17). This process was even faster if a high MW fraction of the extract was supplemented with NADH (not shown). Moreover, small peaks corresponding to reduced Pt(II) complex **1** and DCF were also seen on the chromatogram after 25 min of incubation with the extract, indicating that **4** can, at least to some extent, undergo reduction. This is in agreement with the fact that DCF is known to be rapidly metabolized, undergoing oxidative metabolism to hydroxy metabolites as well as conjugation to glucuronic acid, sulfate, and taurine.^39^ However, due to the complex intracellular environment consisting of many enzymes, identifying the product(s) is a complicated problem whose detailed study is beyond the scope of this work and deserves a separate study.

### Effect on Cancer Cells’ Growth and Viability

The antiproliferative activity of the new compounds toward cancer cells was tested in a panel of six human cancer cell lines. Table 1 shows IC_50_ (concentration of compound that causes death in 50% of cells) values determined using an MTT assay after a 72 h treatment. As indicated, the platinum(II) precursor **1** demonstrated potent activity with submicromolar IC_50_ values, in agreement with the already published data.^2,3,10^ The oxidation of this complex to Pt(IV) analogue **2** resulted in a significant decrease in activity. However, the presence of one or two DCF axial ligand(s) led to a gradual return of biological activity up to the level of the original Pt(II) precursor, although DCF itself showed very little activity. In order to assess the effect of the ethylenediamine linker connecting the DCF to the platinum unit, enDCF was also included as a control in these studies. As indicated, enDCF was significantly (ca 6–8 times) more effective than DCF. The increased activity of enDCF compared to DCF can result from the fact that DCF is negatively charged at biological pH (p*K*_a_ = 4.15) which can significantly reduce its cellular uptake compared to that of enDCF. Nevertheless, the activity of enDCF was still markedly lower than that of Pt complexes **1–4**. All Pt complexes tested in this work were significantly more active than clinically used cisplatin, and, importantly, they were able to overcome cisplatin-induced resistance in A2780cisR ovarian cancer cells. This suggests that the mechanism underlying the biological action of these complexes is at least partially different from that of cisplatin, allowing the compounds to overcome the resistance mechanisms acting in the case of cisplatin.

**Table 1:** IC_50_ Values (Mean ± SD, μM)^a^ Determined for the Investigated 56MeSS Complexes, DCF, enDCF, and Cisplatin by MTT after 72 h of Incubation

complex	HeLa	MDA-MB-231	MCF-7	HCT-116	A2780	A2780cisR	MRC-5	SI
**1**	0.37 ± 0.01	0.24 ± 0.03	0.7 ± 0.1	0.08 ± 0.01	0.19 ± 0.04	0.06 ± 0.01	0.17 ± 0.04	1.2
**2**	2.9 ± 0.7	1.5 ± 0.4	5.9 ± 0.5	1.6 ± 0.4	0.39 ± 0.01	0.40 ± 0.08	2.1 ± 0.4	2.5
**3**	0.6 ± 0.1	0.4 ± 0.1	0.4 ± 0.1	0.20 ± 0.03	0.12 ± 0.02	0.13 ± 0.02	0.7 ± 0.1	3.1
**4**	0.31 ± 0.08	0.29 ± 0.04	0.33 ± 0.05	0.18 ± 0.05	0.05 ± 0.01	0.08 ± 0.02	0.32 ± 0.08	2.6
cisplatin	15 ± 3	22 ± 2	14 ± 3	8 ± 1	3.5 ± 0.6	20 ± 2	7.3 ± 0.9	0.8
DCF	250 ± 22	187 ± 15	259 ± 17	211 ± 10	238 ± 19	138 ± 8	445 ± 36	2.1
enDCF	30 ± 2	32 ± 3	37 ± 4	27 ± 4	ND	ND	58 ± 6	1.8

a Data represent mean ± SD from at least three independent experiments. SI—average selectivity index calculated as IC_50_ (MRC-5)/average IC_50_ (cancer cells). ND = not determined.

In addition to the tumor cell lines, the non-cancerous human fibroblasts MRC-5 were also included in the MTT experiment. As shown in Tables 1 and 2, the SIs determined for Pt(IV) complexes **2–4** were noticeably higher than those obtained for Pt(II) complex **1** in all cancer cell lines except HCT-116 and, importantly, than SIs determined for clinically used cisplatin in all investigated cancer cell lines. This indicates that the selectivity for cancer cells (particularly for ovarian A2780 cells) over normal lung fibroblasts (MRC-5) is improved for Pt(IV) prodrugs vs the parental Pt(II) complex, and it is slightly better for complexes derivatized with DCF ligand(s).

**Table 2:** Selectivity Indices Calculated for Each Tumor Cell Line^a^

compound	HeLa	MDA-MB-231	MCF-7	HCT-116	A2780	A2780cisR
**1**	0.46	0.71	0.23	2.13	0.89	2.80
**2**	0.74	1.48	0.37	1.35	5.49	5.35
**3**	1.06	1.86	1.63	3.35	5.58	5.15
**4**	1.03	1.10	0.97	1.78	6.40	4.00
cisplatin	0.50	0.33	0.53	0.88	2.07	0.37
DCF	1.78	2.38	1.72	2.11	1.86	3.22
enDCF	1.93	1.81	1.57	2.15	ND	ND

a SIs were calculated as IC_50_ (MRC-5)/IC_50_ (individual cancer cell line).

To further verify that preferential cancer cell killing occurs with the Pt(IV) prodrugs derived from complex **1** compared to non-cancerous cells, cancerous HCT-116 and non-cancerous MRC-5 cells were co-cultured and treated with individual agents at concentrations corresponding to their IC_50_ values (Table 1). The selective killing of cancerous cells by Pt(IV) complexes **2–4** was demonstrated based on different morphology (shape, structure, form, and size) of cancerous HCT-116 (epithelial, tile-like morphology) and non-cancerous MRC-5 cells (fibrous) morphology (see the different morphologies of HCT-116 and MRC-5 cells on Figure S18), thus enabling their facile visual identification. Figure S18 shows shots of the co-cultures taken after 72 h incubation with Pt(IV) derivatives of Pt(II) complex **1**. In the control dish, cancerous HCT-116 cells of epithelial, tile-like morphology occupied the surface along with fibrous non-cancerous MRC-5 cells. Furthermore, in the wells containing cells treated with Pt(IV) complexes **2–4**, the number of cancerous HCT-116 cells markedly decreased due to the killing of these cells by complexes **2–4**, in contrast to the number of non-cancerous MRC-5 cells. In other words, the results shown in Figure S18 confirm that Pt(IV) complexes **2–4** selectively induced cell death in human cancer HCT-116 cells but not in normal, healthy cells, even when they were co-cultivated together.

### Accumulation in Cells

The biological activity of anticancer platinum complexes is conditioned by their effective uptake through the cell membrane and their intracellular accumulation. Therefore, the intracellular concentrations of platinum from the investigated Pt(II) and Pt(IV) complexes were determined after the cells were exposed to the complexes at various concentrations and incubation periods and compared to those found for Pt(II) precursor **1** and cisplatin. Moreover, log *P* values of Pt complexes were also determined to assess the possible correlation between the lipophilicity of the Pt complexes and their cellular uptake. The results are summarized in Table 3.

**Table 3:** Amount of Platinum Taken up by HeLa Cells Within 24 h (at 0.1 μM Complex Concentration), or 6 h (1 and 10 μM Complex Concentration), and Log *P* Values of the Investigated Complexes

	cellular uptake (ng Pt/10^6^ cells)			
complex	0.1 μM/24 h	1 μM/6 h	10 μM/6 h	log *P*^a^
**1**	4.4 ± 0.5	13 ± 3	72 ± 2	–1.7 ± 0.2
**2**	2.2 ± 0.2	6.2 ± 0.3	47 ± 2	–2.1 ± 0.2
**3**	3.6 ± 0.2	9.6 ± 0.8	61 ± 8	–1.7 ± 0.1
**4**	3.9 ± 0.3	11 ± 2	82 ± 7	–1.4 ± 0.1
cisplatin	0.3 ± 0.1	2.3 ± 0.3	10 ± 3	–2.3 ± 0.3

a Log *P* (octanol/water) values for the tested platinum compounds determined by the “shake-flask” method.

As indicated in Table 3, oxidation of the Pt(II) complex **1** to its Pt(IV) derivatives resulted in a slight impairment of the transport of platinum into the cells. The amount of platinum taken up by HeLa cells incubated with Pt(IV) dichlorido complex **2** was lower than that from the parental Pt(II) analogue **1**, which correlated with a more negative log *P* value [less lipophilicity determined for Pt(IV) complex **2** (Table 3)]. However, the presence of one or two DCF ligand(s) in the Pt(IV) prodrug **4** renders the complex more lipophilic compared to the Pt(IV) complex **2** and, consequently, the amount of platinum associated with cells increased (Table 3). Thus, the contribution of the DCF moiety to the cellular uptake of the investigated complexes is evident.

A thorough inspection of the data in Tables 1 and 3 revealed the correlation between log *P* values, amount of intracellular Pt, and antiproliferative activity (IC_50_). However, while the cellular uptake of complexes **3** and **4** containing DCF ligand(s) is only 1.3–1.6 and 1.7–1.8 fold, respectively, of the cellular uptake of Pt(IV) complex **2**, the antiproliferative activity increased 4.5- and 9-fold, respectively. These comparisons suggest that contributions of the DCF moiety to the overall activity of Pt(IV) derivatives of Pt(II) complex **1** other than a mere increase of cellular uptake should also be considered.

### Effect on Cytoskeleton Proteins

**Figure 2:**
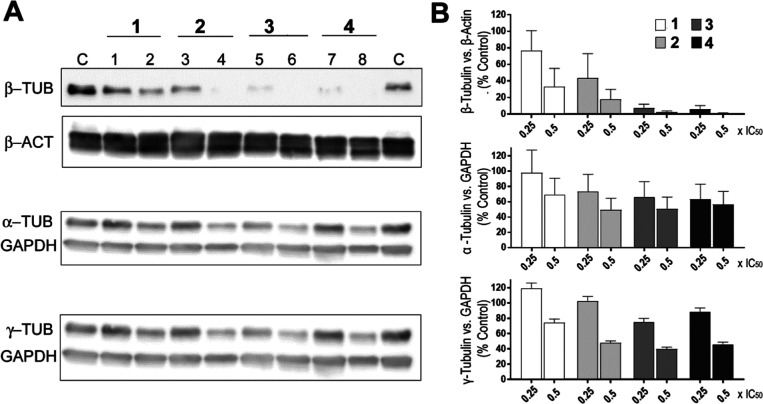
Western blotting analyses of tubulin in protein extract from HeLa cells treated with the investigated Pt complexes. (A) Representative membranes. Cells were treated for 24 h with the indicated complexes at concentrations corresponding to 0.25× IC_50,72h_ (lanes 1, 3, 5, and 7) or 0.5× IC_50,72h_ (lanes 2, 4, 6, and 8). Proteins from control, untreated cells were loaded to lane C. (B) Quantitative data evaluation; bars represent mean ± SD from two independent experiments.

As mentioned in the introductory part, Pt(II) complex **1** is a multimodal complex affecting various cellular targets and processes. Among them, an impact on the cytoskeleton, particularly tubulin, plays a considerable role.^5,40^ As shown in Figure 2, all Pt(IV) complexes tested in this work efficiently reduced the number of tubulin proteins in the extracts of HeLa cells treated with Pt(IV) complexes **2–4**; the effect was most pronounced in the case of the β-tubulin subunit. Notably, the presence of DCF in Pt(IV) complexes **3** and **4** containing DCF axial ligand(s) was reflected in the higher potency of the two complexes to reduce the amount of tubulin as compared to complexes **1** and **2** containing no DCF ligand. This result can be interpreted to mean that although the Pt-part of Pt(IV) complexes **3** and **4** is responsible for reducing the amount of tubulin in the extracts of HeLa cells treated with these complexes, DCF also makes an indispensable contribution to this activity. This conclusion is corroborated by a recent finding that free DCF inhibits microtubule polymerization by direct binding to tubulin.^34^ Thus, the activity of Pt(IV) complexes **3** and **4** containing DCF axial ligands appears to reflect a combination of the effects of both components.

The Pt(IV) complexes **3** and **4** were designed to combine platinum moiety with bioactive ligand(s) DCF, which itself has biological activity, thereby providing dual or multiple mechanisms of action. Therefore, further experiments were aimed at clarifying how the presence of DCF axial ligand(s) in Pt(IV) complexes **3** and **4** contributes to their biological action.

### Effect on Glycolysis

**Figure 3:**
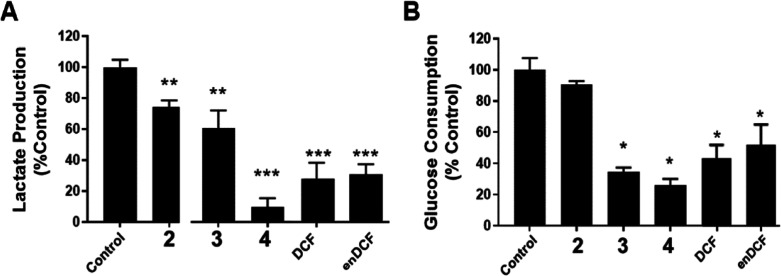
(A) Lactate production. Relative lactate concentration in medium following a 6 h treatment of HeLa cells with the investigated compounds at concentrations corresponding to 5× IC_50_. (B) Glucose consumption by Hela cells treated for 24 h with Pt(IV) prodrugs or free DCF at concentrations corresponding to the IC_50_ values (Table 1). Data represents mean ± SD, *n* = 2–4; asterisks indicate a statistically significant difference from the untreated control (*p < 0.01, **p < 0.005, ***, ***p < 0.001).

DCF was shown previously to target glucose metabolism in cancer cells and, consequently, their proliferation by blocking lactate secretion,^32^ thus reverting the Warburg effect. Therefore, the effect of Pt(IV) complexes **3** and **4** containing DCF axial ligand(s) on lactate transport and glucose metabolism was investigated. As shown in Figure 3A, the concentration of lactate excreted by HeLa cells into media was considerably reduced by treatment with Pt(IV) complexes **3** and **4** containing DCF ligands; the effect was dependent on the number of coordinated DCF molecules. This effect was, as expected, accompanied by a reduction in glucose consumption (Figure 3B) since intracellular lactate, which cannot be excreted into the external environment, effectively inhibits glycolysis. In agreement with the literature data,^32^ similar effects were also observed for free DCF, however, at concentrations markedly higher (380–770 times). Importantly, there was no significant difference in the effects of DCF and enDCF, if applied in their equitoxic concentrations (Figure 3).

### Effect on Mitochondrial Membrane Potential

**Figure 4:**
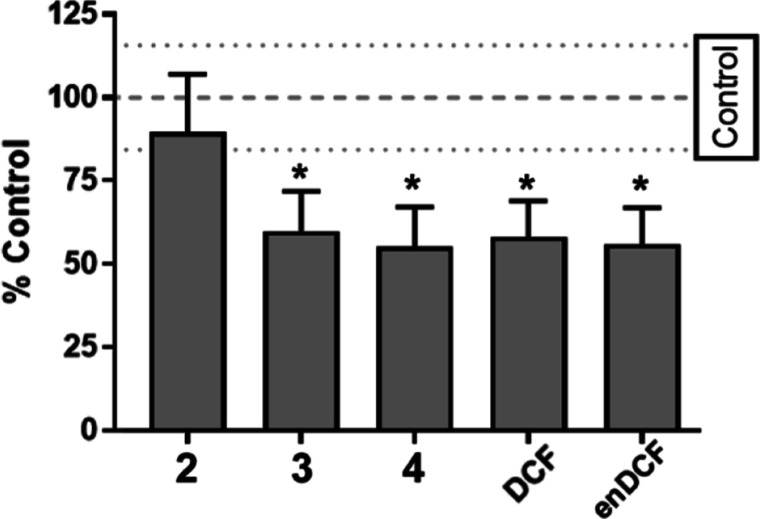
Changes in the mitochondrial membrane potential Δψ_m_ in HeLa cells. The cells were treated with Pt compounds at the concentrations corresponding to 10× IC_50_ for 1 h and subsequently stained with TMRE. The fluorescence was measured by flow cytometry. Data represents mean ± SD from three independent experiments, *p < 0.01.

Changes in the mitochondrial transmembrane potential Δψ_m_ is a parameter frequently studied as its decrease is associated with cell death. As DCF is known to reduce the Δψ_m_,^41,42^ the possible mitochondrial membrane hypopolarization was assessed in tumor cells treated with the tested compounds. Quantitative analysis of tetramethylrhodamine methyl ester (TMRE)-stained HeLa cells revealed a significant (*p* < 0.01) decrease in TMRE fluorescence (proportional to Δψ_m_) in cells treated with both Pt(IV) complexes **3** and **4** bearing one or two axial DCF(s), respectively, compared to untreated control cells (Figure 4). Free DCF, used as a positive control, induced approximately the same effect if applied in its equitoxic concentration (i.e., in the concentration ca. 340- or 770-fold higher than those used for the treatment with **3** or **4**, respectively). The same effect was also found for enDCF in the concentration equitoxic to those used for DCF and Pt compounds (Figure 4).

Notably, the effect of the Pt(IV) complex containing two Cl instead of DCF ligands (complex **2**) showed an insignificant impact on the Δψ_m_, indicating that the DCF rather than the platinum moiety is responsible for the potency of **3** or **4** to reduce the Δψ_m_. Thus, these results suggest the ability of Pt^IV^56MeSS-DCF conjugates to collapse mitochondrial membrane potential in cancer cells due to the presence of metabolically active DCF ligands.

### Effect on COX-2 Expression

In addition to the impact on glucose metabolism and mitochondrial activity, DCF has also shown effects associated with its anti-inflammatory action due to the inhibition of COXs,^43,44^ particularly COX-2.^45^ The effects of Pt(IV) complexes containing DCF on COX-2 expression were therefore studied by Western blotting.

**Figure 5:**
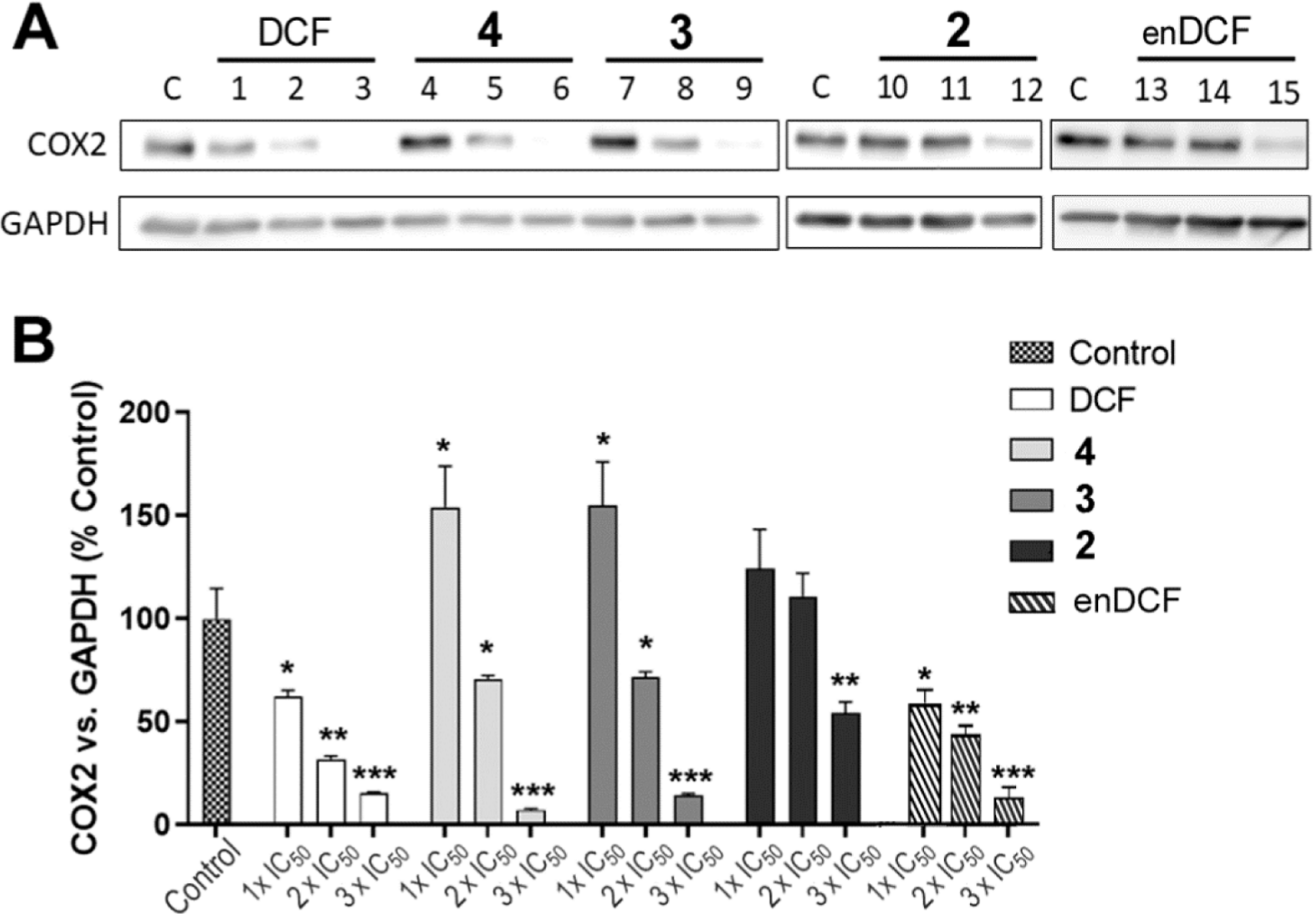
(A) Western blot images of COX-2 and glyceraldehyde-3-phosphate dehydrogenase (GAPDH) levels in HeLa cells non-treated (C) or treated with the tested compounds for 24 h at concentrations corresponding to 1× IC_50_ (lanes 1, 4, 7, 10, and 13), 2× IC_50_ (2, 5, 8, 11, and 14) and 3× IC_50_ (3, 6, 9, 12, and 15). (B) Quantitative evaluation of Western blotting data; the relative expression of COX-2 normalized to GAPDH. Data represents the mean ± SD of two experiments. The stars indicate the statistical significance of difference vs control determined with the Student *t*-test (*p < 0.05, **p < 0.01, ***p < 0.001).

As indicated in Figure 5, both DCF-containing Pt(IV) complexes **3** and **4** were able to reduce the intracellular level of COX-2 protein; the effect was concentration dependent. Similar to the above-described results, free DCF was also active in this respect, as previously published^45^ but only at a concentration more than two orders of magnitude higher; it is of note that free enDCF showed a similar effect. Interestingly, Pt(IV) complex **2** containing no DCF ligand was markedly less effective, suggesting an essential role of DCF ligands in complexes **3** and **4** in their ability to reduce the intracellular level of COX-2 protein. Additionally, the results showed no significant quantitative difference between the effects of complexes **3** and **4**. Since equitoxic concentrations were used, this suggests that inhibition of COX-2 expression contributes approximately equally to the resulting activity of both complexes.

### Inhibition of COX Activity

**Figure 6:**
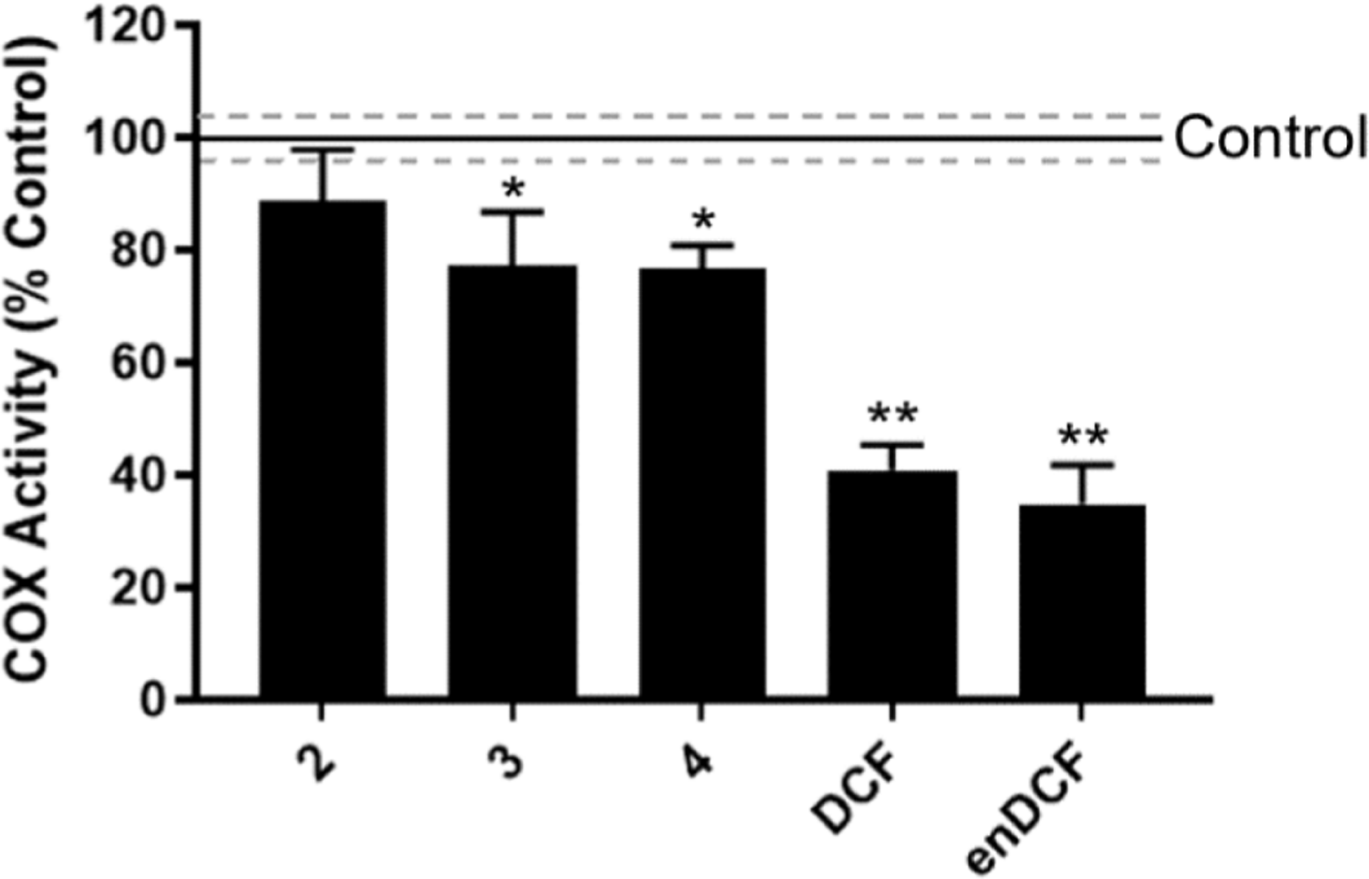
COX-activity in HeLa cells treated with the tested compounds for 24 h at concentrations corresponding to 1× IC_50,72h_, as determined by a COX Activity Assay Kit (Abcam). The results are expressed relative to the COX activity of untreated control. The graph shows mean values ± SD, *n* = 3; asterisks show the statistical difference from the control (*p ≤ 0.05, **p ≤ 0.001).

Previous results showed that the studied Pt(IV) complexes, as well as free DCF ligand, can influence the level of COX-2 expression. To also determine the extent to which the studied substances affect COXs by directly inhibiting the enzyme activity, COX activity was assayed in HeLa cells. For this purpose, cells were treated with concentrations of Pt-complexes that have been shown not to reduce the level of COX-2 protein (IC_50,72h_, Figure 5B). As reported in Figure 6, Pt(IV) complexes **3** and **4** slightly but significantly inhibited COX-2 activity compared to the control, untreated cells. Since **3** and **4** do not decrease the level of COX enzyme under the experimental conditions, they even rather increase it, the reduction of enzymatic activity cannot be attributed to this effect. So, the result clearly shows that Pt(IV) complexes bearing DCF ligand(s) are able to directly inhibit the catalytic activity of COXs. Importantly, DCF has been shown to bind in the active site of COX-2 in a binding mode with its carboxylic acid moiety hydrogen-bonded to Ser-530 and Tyr-385.^46^ It might suggest that DCF is, at least partially, cleaved out from the complex in the intracellular environment so that the carboxylic group is available for binding to the active site of the enzyme and resulting inhibitory effect. Inhibition of COX activity was also observed for both free DCF and enDCF, although to a greater extent than for **3** and **4**, in agreement with higher impact of DCF and enDCF on COX expression.

### Impact on Metastatic Properties

**Figure 7:**
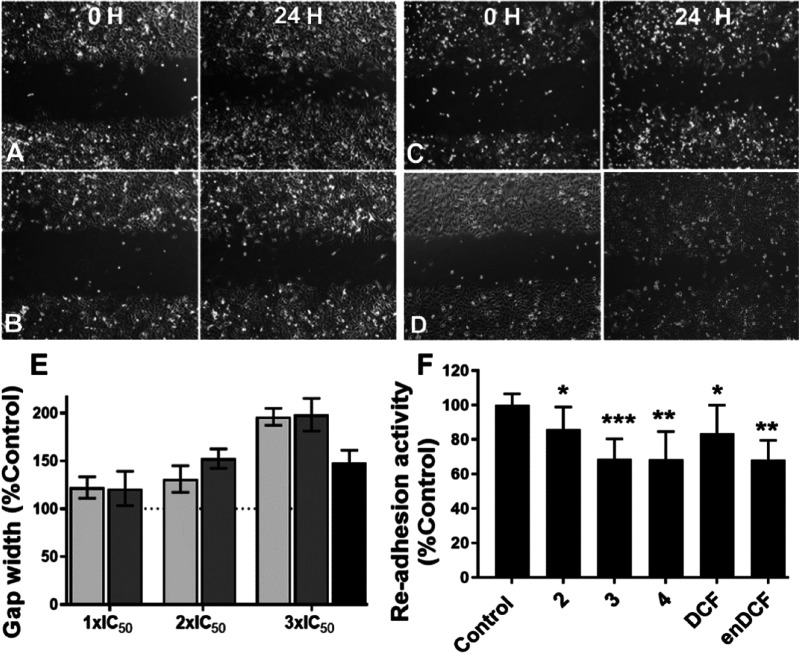
(A–E) Wound healing assay. The monolayer of HeLa cells was scratched with a 10 μL tip, and a starving medium containing the tested complexes was added. The shots were taken at times 0 and 24 h. (A) Control, non-treated cells; (B) cells treated with complex **3**; (C) cells treated with complex **4**. (D) Cells treated with complex **2**; the concentrations of the complexes in the samples shown in Figure 6B–D corresponded to 3× IC_50_ values. (E) Evaluation of wound healing assay. Bars represent gap size vs. Control (100%), following a 24 h incubation with the investigated compounds. Light gray bars: complex **3**; dark gray bars: complex **4**; black bar: complex **2**. (F) Re-adhesion assay. HeLa cells were treated with the tested compounds at concentrations corresponding to 10× IC_50_ (MTT, 72 h) for 1 h. Following trypsinization, the cells were resuspended in Dulbecco’s modified Eagle medium (DMEM), counted, left to reconstitute adhesive membrane proteins for 30 min, and re-seeded at the density of 3× 10^4^ cells/100 μL/well. After 30 min of incubation at 37 °C, the re-adhesion activity was determined as described in the Experimental Section. Data represents mean ± SD, *n* = 8; *p < 0.05, **p < 0.01, ***p < 0.001.

COX isoform COX-2 is frequently expressed in many types of cancer and induces CSC-like activity, promoting apoptotic resistance, proliferation, angiogenesis, inflammation, invasion, and metastasis of cancer cells.^47,48^ An initial step in tumor metastasis is the invasion of cancer cells into surrounding tissue and the vasculature, which requires the chemotactic migration of cancer cells.^49,50^ Migratory and invasive properties of tumor cells are closely connected with their adhesivity.^51^ Therefore, the migration and re-adhesion activities of HeLa cancer cells treated with Pt(IV) complexes **2–4** and both free DCF and enDCF were evaluated to assess their effect on the metastatic potential of tumor cells. The results in Figure 7A–D show that the treatment with both DCF-containing Pt(IV) complexes **3** and **4** reduced the ability of HeLa cells to close artificial wounds (scratches) in monolayers when compared to untreated control cells. Thus, both complexes diminish the migration activity of cells in a concentration-dependent manner; the effect is quantitatively the same if the compounds are applied at equipotent concentrations (multiples of IC_50,72h_). In contrast, Pt(IV) complex **2** containing no DCF ligand was less efficient in inhibting artificial wound closing.

Similar to migration, both Pt(IV) prodrugs **3** and **4** containing DCF ligand(s) lower the ability of HeLa cells to re-adhere to the growth surface (Figure 7E). This effect was more pronounced for the DCF-containing complexes compared to the Pt(IV) complex bearing two chlorides (although applied in equitoxic doses), suggesting the contribution of DCF ligands to this effect.

### Hallmarks of Immunogenic Cell Death

An escape from immune surveillance of cancer cells is a crucial mechanism of cancer progression and metastatic dissemination and creates a serious obstacle to successful cancer treatment.^52^ Thus, a combination of chemotherapy with strategies aiming to induce tumor-specific immunity that would control the growth of residual tumor cells represents a promising approach.

Recently, it has been shown both in vitro and in vivo^53−60^ that in contrast to cisplatin, oxaliplatin and its analogues induce immunogenic cell death (ICD) and thereby synergistically potentiate antitumor effects. It has also been shown that even minor changes of the 1*R*,2*R*-diaminocyclohexane ring of the oxaliplatin molecule may have an important impact on its immunomodulatory activity.^55^ These observations suggest that the cyclohexane ring of oxaliplatin is a determining factor in the mechanism by which oxaliplatin induces ICD in tumor cells. The unconventional Pt(IV) complexes **2–4** tested in this work, as well as the parental Pt(II) complex **1**, contain a 1,2-diaminocyclohexane ring (although in a 1*S*,2*S* configuration). Moreover, NSAIDs have also been shown^61^ to induce hallmarks of ICD in cancer cells. Therefore, it was attractive to test whether the new Pt(IV) prodrugs **2–4** could stimulate biochemical processes characteristic of ICD.

ICD is accompanied by the exposure and release of numerous damage-associated molecular patterns (DAMPs), which altogether confer a robust adjuvanticity to dying cancer cells, as they favor the recruitment and activation of antigen-presenting cells.^62^ ICD-associated DAMPs include surface-exposed calreticulin (CALR), as well as secreted ATP, and high-mobility group box 1 (HMGB1).^63^

**Figure 8:**
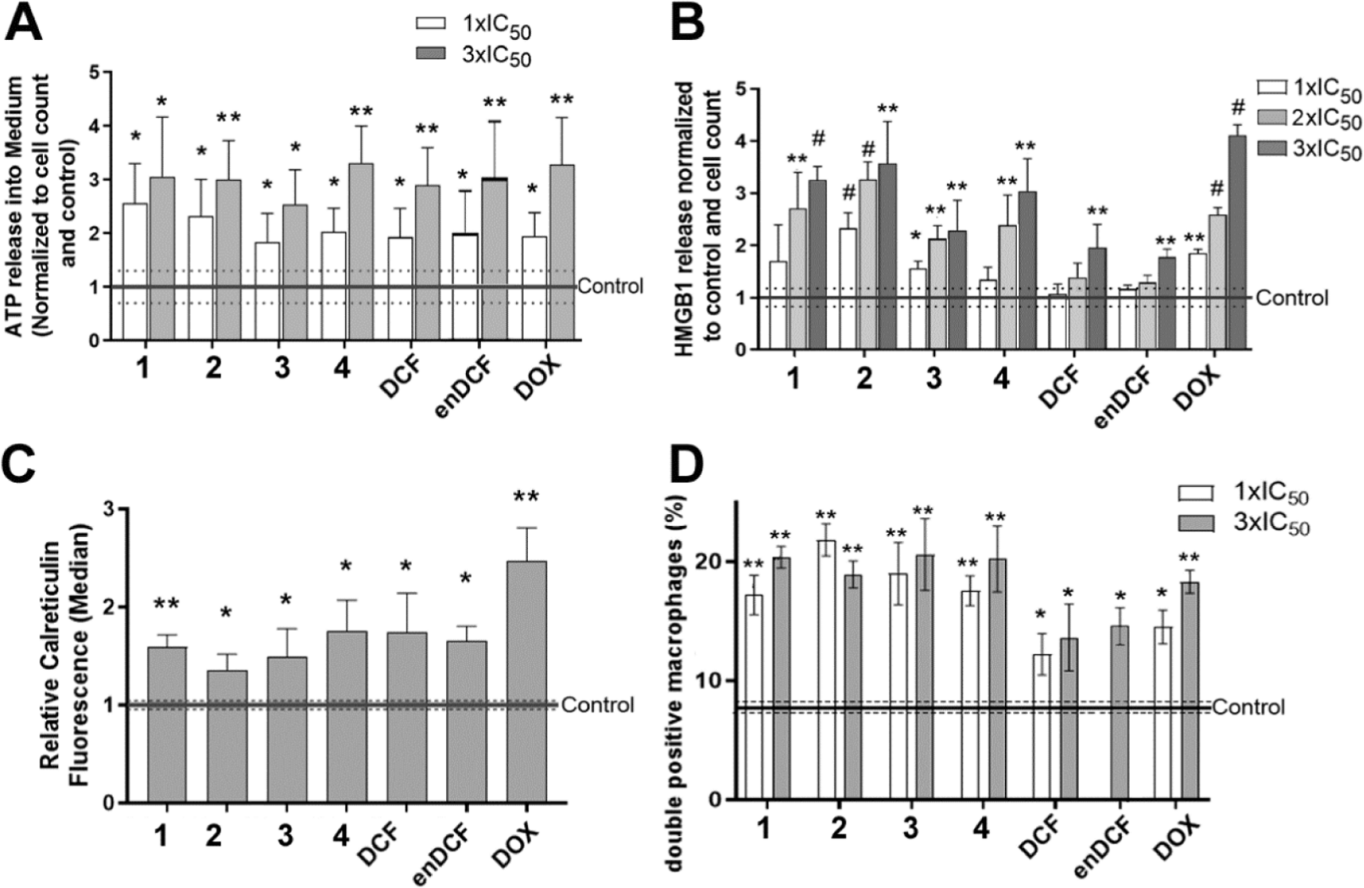
Hallmarks of ICD. (A) Extracellular ATP levels in HeLa cell supernatants. HeLa cells were treated with the tested compounds at concentrations corresponding to 1× IC_50_ and 3× IC_50_ values. (B) Extracellular level of HMGB1. HeLa cells were treated with the tested compounds at concentrations corresponding to 1× IC_50_, 2× IC_50,_ and 3× IC_50_ values. (C) Calreticulin exposure to the cell surface. HeLa cells were treated with the tested compounds at concentrations corresponding to 3× IC_50_. The surface calreticulin exposure was determined in 4′,6-diamidino-2-phenylindole dihydrochloride (DAPI)-negative cell population. Relative fluorescence intensity was calculated as the median fluorescence of the population of treated cells/median fluorescence of untreated control cells. The data in panels A, B, and C are shown as a mean of 4–5 measurements ± SD. Statistical significance from untreated control was determined with the Student *t*-test (*p ≤ 0.05, **p ≤ 0.01, and ^#^*p* ≤ 0.001). (D) Quantitative evaluation of phagocytosis of HeLa cells treated with the investigated compounds by human Thp-1/PMA macrophages (analyzed by flow cytometry). Hela cells were treated for 24 h with the tested compounds at concentrations corresponding to 1× or 3× IC_50_,_72h_. The full horizontal line indicates the level of phagocytosis of the untreated control cells. Error bars are the SEMs calculated from three independent experiments. The stars denote a significant difference from untreated control (*p ≤ 0.05, **p ≤ 0.01) as calculated by the nonparametric Student *t*-test.

The results demonstrate the ability of the tested Pt(IV) complexes **2–4** to effectively provoke ATP (Figure 8A) and HMGB1 (Figure 8B) secretion from cancer cells. This effect was concentration-dependent and quantitatively similar to that induced by the well-known ICD inducer doxorubicin (taken as positive control). Similar effects were also observed when externalization of intracellular calreticulin was followed (Figures 8C and S19). Interestingly, the parental compounds Pt(II) complex **1** and DCF or enDCF were effective as well, although the effect of DCF or enDCF was evident only at significantly higher (80–600 fold) concentrations. Thus, the activity of Pt(IV) complexes **2–4** leading to the release of DAMPs can be attributed to the effects of the Pt part of the tested Pt(IV) complexes, although an effect of the DCF ligand cannot be ruled out.

Additionally, an essential feature of ICD is that ICD inducers should also increase tumor cell phagocytosis.^64^ A stimulation of immunogenic phagocytosis belongs to the most critical hallmarks of ICD.^65^ To verify whether this aspect of ICD is also included in the effects of the investigated Pt(IV) complexes, a human in vitro model was used for testing the ability of macrophages to recognize human cancer cells treated with the tested compounds. For this purpose, we used human cervical cancer HeLa cells and human monocytic Thp-1 cells activated into macrophages by differentiating with phorbol 12-myristate 13-acetate. Hela cells were treated with the investigated compounds at their concentrations corresponding to 1× or 3× IC_50,72h_ and incubated for 24 h. Then, both cell populations were stained with either green (ThP-1/PMA cells) or red (HeLa cells) tracker dyes and co-incubated for 2.5 h at the ratio of 1:3 (effector/target). Phagocytosis was classified by the occurrence of double-positive macrophages.

As shown in Figures 8D and S20, the treatment with all investigated Pt(IV) complexes increased tumor-cell phagocytosis markedly (2–2.5 fold). Notably, doxorubicin investigated in this study as positive control exhibited slightly less pronounced levels of phagocytosis, suggesting very effective stimulation of immune cells by the cancer cells treated with the Pt-complexes. Similar to DAMPs production, the parental Pt(II) complex **1** was roughly as effective as its Pt(IV) analogues **2–4**. Interestingly, clinically used oxaliplatin, although it induces ICD and key pro-phagocytic signals, does not promote tumor cell phagocytosis.^66−68^ This favors the new Pt–DCF complexes over the clinically used oxaliplatin in terms of their ability to induce ICD in cancer cells.

The extent of phagocytosis is a major indicator of stimulation of the organism’s immune response and may predict the in vivo efficiency of the agents inducing ICD.^69^ The observation that Pt^IV^56MeSS-based complexes promote tumor cell phagocytosis significantly entitles us to suggest that these complexes can be considered prospective drugs, active in inducing immunity against tumor cells better than other metal-based complexes used clinically. Interestingly, antiglycolytic treatment (such as that with DCF) of cancer cells is known to trigger an antitumor immune response as well via enhancement of the antitumor immune activity of T-cells.^70^ Thus, the DCF ligand can contribute to the stimulation of other components of antitumor immunity due to its effect on glucose metabolism. The Pt(IV) complexes prepared and tested within this work may thus represent candidate prodrugs combining metabolic effects on cancer cells with an activation of the immune response against cancer, which determines the long-term success of anticancer therapies.

## Conclusions

In this work, we present new Pt^IV^56MeSS derivatives bearing one or two molecules of DCF as axial ligand(s). These complexes were designed to prepare prospective Pt-based prodrugs exhibiting mechanisms of action typical for Pt^II^56MeSS complexes and DCF simultaneously. We demonstrate the superior antiproliferative activity of the new Pt(IV) complexes containing DCF axial ligand(s) against a panel of cancer cell lines of various origins, particularly in the case of Pt(IV) complex **4** (containing two axial DCF ligands), which achieved comparable or even better activity than the parental Pt(II) complex **1**. In contrast to complex **1** and cisplatin, the new Pt(IV) complexes show improved selectivity to cancer over the non-cancerous cells.

The coordination of Pt^IV^56MeSS and DCF into one Pt(IV) prodrug also provides an advantage of broadly multimodal anticancer effect, embracing both the effects of the platinum moiety (impact on tubulin cytoskeleton, DNA interaction) and the activities characteristic of DCF (inhibition of glycolysis, COX-2 inhibition resulting in anti-inflammatory and anti-migratory properties), not achievable by mixtures of the single agents (Pt/DCF = 1:1 or 1:2). These results also confirm the hypothesis that when DCF is coordinated in the axial position(s) of the investigated Pt(IV) complexes, it enters the cells simultaneously in one molecule with the Pt moiety. Interestingly, biological characteristics of DCF were also seen for enDCF if applied in equipotent concentration. This suggests that the presence of the ethylenediamine linker, if it remains attached to DCF, does not qualitatively affect the resulting biological properties. Moreover, the cleavage of the peptidic bond in the acidic environment of the lysosome or endosome, as well as its enzymatic hydrolysis by peptidases or proteases in the intracellular environment, can also be reasonably assumed.

On the basis of the data previously published on similar complexes,^30^ we hypothesize that upon intracellular accumulation of Pt(IV) complexes, the DCF ligand might be cleaved off either by reduction or enzymatic cleavage of the Pt(IV) complex. Thus, besides the platinum moiety, free DCF molecules would be released, which could promote antiproliferative activity also through the inhibition of lactate transporters, resulting in blockage of the glycolytic process and impairment of mitochondrial potential. On the other hand, it cannot be entirely excluded that improvement and enrichment of the activity of the original parent Pt(II) complex **1** are achieved without the release of free DCF molecules from the Pt(IV) complexes. This multifactorial mechanism of action, affecting a number of different biological/biochemical pathways and processes, may represent a great advantage as it is very difficult for tumor cells to develop resistance against so many different mechanisms acting simultaneously.

Clinically apparent tumors evolve mechanisms to evade immune elimination. Therefore, the induction of the immune system to recognize and eliminate malignant cells represents an important task in anticancer strategies. A combination of chemotherapy with immunotherapeutic strategies aiming to induce tumor-specific immunity is challenging because chemotherapy is generally considered to be immunosuppressive. We show in this work that the Pt(IV) complexes combining Pt(II) complex **1** and DCF effectively induce hallmarks of ICD in cancer cells and promote phagocytosis of tumor cells. In this respect, the coordination of DCF to Pt^IV^56MeSS could pave the way for the development of new, therapeutically relevant chemotherapeutics that would be able to overcome resistance to chemotherapy and be capable of preventing tumor reoccurrence through the stimulation of anticancer immunity.

## Experimental Section

### NMR Reduction Studies

One equivalent of the complex was dissolved in 500 μL of PBS using D_2_O for a ^1^H NMR experiment performed on a Bruker AVANCE 400 MHz NMR spectrometer, with 50 dummy scans (approx. 5 min) to allow the sample to reach 37 °C before 128 scans were taken. The sample was removed, and the reducing agent was quickly added. Then the NMR measurement was taken again (50 dummy scans followed by 128 scans). The NMR was subsequently set up to measure one 128-scan experiment after another for 4 h, at which point the sample was removed, stored, and scanned again after 24 and 48 h. Three reduction studies were undertaken using complex **4** (7.0 mg) together with ascorbic acid (2.5 mg, 3 equiv), complex **4** (6.1 mg) together with glutathione (GSH) (7.0 mg, 5 equiv), and complex **4** (15.4 mg) together with ascorbic acid (17.5 mg, 10 equiv).

### Electrochemistry

Electrochemical measurements were performed using an Autolab PGSTAT 302, Metrohm. Cyclic voltammetry was carried out at a scan rate of 50 mV s^–1^ over the range 0.7 to −1.5 V, using a glassy carbon working electrode, a platinum wire auxiliary electrode, and Ag/AgCl, KCl, *c* = 3 M reference electrode at 25 °C. The samples were prepared as 1 mM solutions in PBS and were deoxygenated with a stream of argon through the solution immediately prior to measurement.

### HPLC Analysis after Incubation with Cell Extract

HeLa cells were cultured in DMEM at 37 °C, 5% CO_2_ until they reached a confluence. Then, the cells were scraped, washed, pelleted by centrifugation, and lysed with ice-cold RIPA buffer supplemented with PMSF, sodium orthovanadate, and a protease inhibitor cocktail. To obtain the high molecular mass fraction of the extract, it was transferred to a centricon (Nanosep 3k Omega, Life Sciences) with a cut-off of 3000 Da and centrifuged at 14,000g for 25 min at 4 °C. The centricon was then turned upside down, and the high molecular mass fraction was collected into an Eppendorf tube. Complex **4**, DCF, or enDCF was incubated with this cell fraction for the indicated time. The reaction products were analyzed by HPLC. Condition of analyses: RP HPLC: gradient 0–100% B in 15 min (A = 0.1% TFA, B = ACN). Symmetry C18 Column, 3.5 μm, 4.6 mm × 75 mm (Waters). HPLC system: Waters 1525 Binary HPLC Pump with 2489 UV/visible dual detector. The detector was set to a wavelength of 280 nm. Before injection, excess proteins were removed by filtering through the column (cut off 3 kDa).

### Cell Lines

The human cervical carcinoma HeLa, human ovarian carcinoma A2780, and human breast adenocarcinomas MDA-MB-231 and MCF-7 were purchased from the European collection of authenticated cell cultures ECACC (Salisbury, UK), human monocytic cell line Thp-1 was purchased from the American Type Culture Collections (ATCC), human colon carcinoma HCT-116 was kindly provided by Dr. M. Brazdova, Institute of Biophysics (Brno, CZ), and cisplatin-resistant human ovarian carcinoma A2780cisR (a cisplatin-resistant variant of A2780 cells) was kindly provided by Professor B. Keppler, University of Vienna (Austria). A2780 and A2780cisR cells were grown in RPMI-1640 medium (Biosera, Boussens, France) supplemented with gentamycin (50 μg mL^–1^, Serva, Heidelberg, Germany) and 10% heat-inactivated FBS (Biosera). The other cells were grown in DMEM medium (high glucose 4.5 g L^–1^, PAA) supplemented with gentamycin (50 μg mL^–1^, Serva) and 10% heat-inactivated FBS (Biosera). The cells were cultured in a humidified incubator at 310 K in a 5% CO_2_ atmosphere and subcultured 2–3 times a week with a desired plating density.

### Antiproliferative Effect in Cancer Cells

MTT ([3-(4,5-dimethyl-2-thiazolyl)-2,5-diphenyl-2*H*-tetrazolium bromide])-based assay was used to evaluate the toxic/antiproliferative effect of the studied compounds on cells. The cells were plated (96-w) at their respective previously determined optimal densities (5 × 10^3^ HeLa, A2780, A2780cisR, MDA-MB-231, and MRC-5 cells/well; 4 × 10^3^ HCT-116 cells/well; and 3 × 10^3^ MCF-7 cells/well) and grown overnight. Cells were treated with the tested compounds in a final volume of 200 μL/well. After 72 h incubation, 10 μL/well of freshly diluted MTT (2.5 mg mL^–1^ in PBS; Calbiochem, Darmstadt, Germany) was added, and the cells were incubated for an additional 2–4 h. The medium was removed, and the insoluble formazan product was dissolved in 100 μL/well DMSO. The absorbance was read at 570 nm (vs 620 nm) using an Absorbance Reader (SUNRISE TECAN SCHOELLER). The read values were converted to the percentage of control. The resulting effect was expressed as IC_50_ values (compound concentration at which the produced signal corresponds to 50% of the control signal). All experiments were made in triplicate.

### Treatment of Co-cultured HCT-116 and MRC-5 Cells with Pt(IV) Complexes

HCT-116 and MRC-5 cells were seeded in the same wells of 6-well plates at densities 1 × 10^4^ (HCT-116) and 2 × 10^4^ (MRC-5). These densities lead to similar cell counts of both cell lines at the time of treatment, approximately 24 h after seeding. The co-cultures were treated with Pt(IV) complexes at their respective IC_50_ concentrations (MTT; 72 h). The images were taken following the 72 h treatment with a Canon EOS 1200D camera attached to an Olympus CKX41 inverted microscope with a 10×/0.25 phase contrast objective.

### Determination of Partition Coefficients

The “shake flask” method was used to measure the partition coefficients (*P*) of platinum compounds. The compounds were dissolved in octanol-saturated water (OSW) containing 200 mM NaCl. Mixtures containing OSW (Pt compounds solutions) and water-saturated octanol (WSO) in a volumetric ratio 1:1 were vortexed for 30 min at room temperature to establish the partition equilibrium. The water and organic phases were separated by centrifugation (3000*g*; 5 min). After careful separation of the layers with a fine-tip pipet, the Pt content in individual phases was determined by flameless atomic absorption spectrometry. The partition coefficients were calculated as the concentration ratio of the compound in the octanol layer to that in the aqueous layer, log *P* = log([Pt]WSO/[Pt]OSW).

### Cellular Accumulation of Platinum

The uptake of the platinum complexes by HeLa cells was determined as the platinum amount per 10^6^ cells. Briefly, 3 × 10^6^ cells were seeded in 100 mm culture dishes and grown overnight. The cells were exposed to the tested complexes at indicated concentrations for indicated periods. After the incubation, the cells were washed extensively with PBS, harvested using 0.25% trypsin, washed with ice-cold PBS (2×), and pelleted. The pellets were digested using the microwave acid (HCl) digestion system (CEM Mars). The platinum quantity taken up by the cells was determined by inductively coupled plasma mass spectrometry (ICP–MS). All experiments were performed in triplicate.

### Lactate Production

HeLa cells were seeded (1 × 10^5^ cells/well) and grown in 12-well plates, treated with platinum agents, and diclofenac for 6 h. Lactate concentration in the culture medium was measured using a colorimetric Lactate Assay Kit (Sigma-Aldrich) following the manufacturer’s instructions. The enzymatic reaction resulted in a colorimetric product proportional to the lactate concentration that was measured at 570 nm with an absorbance reader (SPARK, Tecan). The amount of lactate in individual samples was determined from a standard curve and expressed as a percentage of non-treated control.

### Glucose Consumption

HeLa cells were seeded (3 × 10^5^ cells/well), grown in 6-well plates and treated with platinum agents and DCF for 24 h. Glucose concentration in the culture medium prior to and after the treatment was determined with an Amplex Red Glucose/Glucose Oxidase Assay Kit (Invitrogen) following the producer’s protocol. Glucose consumption was normalized to final cell counts. The glucose amount consumed by the non-treated control was taken at 100%.

### Changes in Mitochondrial Membrane Potential

HeLa cells were seeded (6-well plates, 3 × 10^5^ cells/well), grown overnight, and then treated with the tested compounds for 1 h. The medium was removed, and a fresh medium containing TMRE (Thermo Fisher Scientific) at a final concentration of 1 nm in 500 μL was added. The cells were incubated for 30 min at 37 °C and then harvested using trypsin. TMRE fluorescence was measured with a fluorescence-activated cell sorting (FACS) Verse flow cytometer (Becton Dickinson), and the data were analyzed using the ModFit LT 4.1 (Verity Software House) software. The experiment was performed in triplicate.

### Western Blotting of COX-2 and Tubulin

HeLa cells were seeded in 60 mm dishes at a density of 3 × 10^5^ cells/dish and cultured for 20 h. The cells were then treated with the tested compounds at concentrations indicated in the text. After 24 h of treatment, the cells were scraped, washed, and pelleted. The pellets were then lyzed with RIPA buffer supplemented with proteinase inhibitors following the manufacturer’s recommendation (1 h on ice), and the extracts were cleared with centrifugation (15,000 rpm/10 min), combined with 2× LBS Buffer (4% SDS, 20% glycerol, 10% 2-mercaptoethanol, 0.004% bromophenol blue, 0.125 M Tris–HCl) and heated for 10 min at 95 °C. 4–20% SDS-PAGE (sodium dodecyl sulfate-polyacrylamide gel electrophoresis) (Mini-PROTEAN TGX Precast Gel) was used to resolve the proteins. After transferring to the polyvinylidene fluoride membrane, the proteins were detected using appropriate antibodies: anti-GAPDH antibody (mouse, Sigma-Aldrich), Anti-COX-2 (rabbit, Abcam), Anti-α-, β-, or γ-tubulin (all rabbit, Abcam), Anti-β-actin (mouse, Abcam), Goat Anti-Rabbit IgG(HRP) (Abcam; HRP = horseradish peroxidase), and Goat Anti-Mouse IgG(HRP) (ThermoFisher Scientific). SignalFire ECL Reagent (A + B) was used as a substrate for HRP, and the luminescence was recorded with Amersham Imager 680. The densities in the images were assessed with the Aida image analysis software.

### COX Activity Assay

HeLa cells were seeded in 6-well plates at a density of 10^5^ cells/well and incubated overnight. The cells were treated with the tested compounds at concentrations corresponding to 1× IC_50_. Following a 24 h treatment, the cells were lyzed with radioimmunoprecipitation assay (RIPA) buffer supplemented with proteinase inhibitors on ice for 10 min. The extracts were cleared with centrifugation (12,000*g*/5 min), and protein content was determined using Bradford assay. COX activity was determined with a COX Activity Assay Kit (Abcam). 6 μg of protein was used in the reaction. The experiment was set up according to the manufacturer’s instructions. The fluorescence (Ex/Em 535/587 nm) was read in a kinetic mode, and the COX inhibition was expressed as a percentage of the activity of the untreated control.

### Wound Healing Assay

HeLa cells were grown close to confluence in 6-well plates in a complete medium, and then the medium was replaced with a serum-free medium [supplemented with 0.1% bovine serum albumin (BSA)], and the cells were incubated for an additional 24 h. The HeLa monolayers were scratched with a 10 μL pipet tip, and the cells were washed twice with PBS to remove peeled cells and treated with tested compounds at indicated concentrations. The scratched areas were shot immediately after the complex addition and then after 24 h with a Canon EOS 1200D camera attached to an Olympus CKX41 inverted microscope with a 10×/0.25 phase contrast objective. Digital images were taken by the QuickPHOTO MICRO 3.1 program (PROMICRA, Prague, Czech Republic) and processed with the TScratch analysis software (ETH Zürich, Switzerland). The cell’s ability to migrate into the open area was expressed as a percentage of control.

### Re-adhesion Assay

HeLa cells were grown in 6-well plates for 24 h. The cells were treated with the tested compounds at concentrations corresponding to 10× IC_50_ values for 1 h. Following the treatment, the cells were trypsinized, washed, and resuspended in fresh serum-free medium, counted, and left at room temperature for 30 min to allow surface receptor reconstitution. Then the cells were seeded in a 96-well plate at a density of 3 × 10^4^ cells/well in 100 μL of media in octuplicate and incubated to adhere at 37 °C for 30 min. The medium containing non-attached cells was removed, and the wells were gently washed twice with PBS. The number of adhered cells was determined with sulforhodamine B (SRB) assay. Briefly, the attached cells were fixed with 10% v/v trichloroacetic acid for 1 h at 4 °C, washed thoroughly with Milli-Q water, air-dried, stained with SRB solution (0.4% w/v in 1% acetic acid) for 30 min at room temperature, washed with 1% acetic acid three times, and air-dried. The bound SRB was dissolved in 10 mM Tris base (pH 10.5), and the absorbance was recorded at 570 nm with an Absorbance Reader (SUNRISE TECAN, SCHOELLER). The data were expressed relative to the untreated control.

### HMGB1 Release

HeLa cells were seeded in 24-well plates at a density of 10^4^ cells/well and grown overnight. The medium was removed, and a fresh medium (DMEM, 1% BSA, no FBS) containing indicated concentrations of tested complexes was added (300 μL). Following a 20 h treatment, medium samples were withdrawn, and the HMGB1 content was determined using an HMGB1 ELISA Kit (IBL international) following the instructions for use. Cell counts in corresponding wells were determined using Automated Cell Counter (Bio-Rad). HMGB1 concentrations were normalized to cell counts and to the value of untreated control. The experiment was performed twice with duplicate readings.

### ATP Secretion

HeLa cells were seeded in 24-well plates at a density of 10^4^ cells/well, grown overnight, and then treated with the tested complexes for 24 h. Aliquots of culture medium samples were withdrawn, centrifugated (500*g*, 3 min), and processed with ATP Bioluminescence Assay Kit CLS II, Roche. Briefly, 50 μL of cell-free medium samples were added to a 96-well flat white plate, and an equal volume of luciferase reagent was added. The luminescence was measured immediately on a plate reader (SPARK, Tecan). The blank (no ATP) was subtracted from the raw data. ATP concentrations were obtained from the standard curve and normalized to cell counts and control. The data are shown as MEAN of 4–5 independent measurements.

### Calreticulin Exposure

HeLa cells were seeded (2 × 10^5^ cells/well) in 6-well plates, grown overnight, and treated with the tested compounds at concentrations corresponding to 3× IC_50_ (MTT, 72 h) for 16–18 h. Following the treatment, the cells were harvested (trypsin), washed with FACS buffer (PBS, BSA 1%, and FBS 2%), and incubated with Alexa Fluor 488 Anti-Calreticulin antibody for 40 min at 4 °C. The buffer was removed (CFG, 300 g, 3 min) and replaced with FACS buffer containing DAPI (1 μg mL^–1^, 20 min), and the cells were analyzed with flow cytometry (BD FACS Verse) and FCS Express 6 (DeNovo Software, Glendale, CA). Calreticulin fluorescence histograms were analyzed in the DAPI-negative (intact membrane) cell population.

### Detection of Phagocytosis

Human monocytic leukemia cells Thp-1 (obtained from American Type Culture Collection, ATCC) were differentiated into macrophages with phorbol 12-myristate 13-acetate (PMA, 100 nM) for 24 h. Hela cells were treated for 24 h with the tested compounds at concentrations corresponding to 1× or 3× IC_50,72h_ and incubated. Then, both cell populations were stained with cell trackers. ThP-1/PMA were stained with CellTracker (green CMFDA; Thermo Fisher Scientific); Hela cells were stained with CellTracker (red CMTPX; Thermo Fisher Scientific). Both cell lines were co-incubated for 2.5 h at the ratio of 1:3 (effector/target), harvested, and fixed for 10 min with 4% formaldehyde. Phagocytosis was evaluated using flow cytometry (BD FACSVerse), and data were analyzed with FCS Express 7 (DeNovo software; Glendale, CA). Samples were analyzed by flow cytometer BD FACS Verse. Phagocytosis was classified by the occurrence of a double-stained cell population of macrophages.

## Supporting Information Available

Detailed description of synthesis, experimental details, NMR spectra and HPLC traces, determination of reduction potentials by CV, effect of incubating complex 4 with HeLa cell extract, morphologies of HCT-116 and MRC-5 cells, amount of Pt associated with DNA isolated from HeLa cells, flow cytometric analysis of calreticulin exposure in HeLa cells, and flow cytometry density plots showing phagocytosis (PDF)
Molecular formula strings and biological data (CSV)

## Abbreviations

BSA: bovine serum albumin
CALR: calreticulin
COX: cyclooxygenase
CSC: cancer stem cell
DACH: diaminocyclohexane
DAMP: damage-associated molecular pattern
DAPI: 4′,6-diamidino-2-phenylindole dihydrochloride
DCF: diclofenac
DMEM: Dulbecco’s modified Eagle medium
DMSO: dimethylsulfoxide
enDCF: diclofenac amide
FACS: fluorescence-activated cell sorting
FBS: fetal bovine serum
GAPDH: glyceraldehyde-3-phosphate dehydrogenase
HMGB1: high-mobility group box 1
IC_50_: concentration of compound that causes death in 50% of cells
ICD: immunogenic cell death
MTT: 3-(4,5-dimethylthiazol-2-yl)-2,5-diphenyltetrazolium bromide
NSAID: non-steroidal anti-inflammatory drug
OSW: octanol-saturated water
PAGE: polyacrylamide gel electrophoresis
PBS: phosphate-buffered saline
SDS: sodium dodecyl sulfate
SI: selectivity index
SRB: sulforhodamine B
TMRE: tetramethylrhodamine ethyl ester
WSO: water-saturated octanol
Δψ_m_: mitochondrial transmembrane potential
