# Supplementary material for: Platinum(IV) Derivatives of [Pt(1S,2S-diaminocyclohexane)(5,6-dimethyl-1,10-phenanthroline)] with Diclofenac Ligands in the Axial Positions: A New Class of Potent Multi-action Agents Exhibiting Selectivity to Cancer Cells
Source: J Med Chem. 2023 Jun 7;66(12):7894–908. doi: 10.1021/acs.jmedchem.3c00269 (PMC10291556; doi:10.1021/acs.jmedchem.3c00269)
Supplement: Supplementary file 1 — jm3c00269_si_001.pdf [file jm3c00269_si_001.pdf]

Supplementary material

Platinum(IV) derivatives of [Pt(1*S*,2*S*-diaminocyclohexane)(5,6-dimethyl-1,10-phenanthroline)] with diclofenac ligands in the axial positions: a new class of potent multi-action agents exhibiting selectivity to cancer cells

Hana Kostrhunova[a], Brondwyn McGhie[b], Lenka Markova[a], Olga Novakova[a],Jana Kasparkova[a,c], Janice Aldrich-Wright[c,*], Viktor Brabec[a,*]

[a] *Czech Academy of Sciences, Institute of Biophysics, Kralovopolska 135, 61265 Brno, Czech Republic*
[b] *School of Science and Health, Western Sydney University, Penrith South DC 1797, NSW, Australia*
[c] *Department of Biophysics, Faculty of Science, Palacky University, Slechtitelu 27, 783 71 Olomouc, Czech Republic*

*Corresponding author e-mail addresses:
brabec@ibp.cz (V. Brabec), J.Aldrich-Wright@westernsydney.edu.au (J. Aldrich-Wright)

Table of Contents

Experimental ………S2

Detailed description of synthesis ……………………………………………………………………………… S3

NMR spectra and HPLC traces (Figures S1-S16) ………………………………………………………………S4

Determination of the reduction potentials by cyclic voltammetry ……………………………………… S13

The effect of incubating complex 4 with HeLa cell extract (Figure S17) ……………………………… S14

Morphologies of HCT-116 and MRC-5 cells (Figure S18) ……………………………………………………S15

The amount of Pt associated with DNA isolated from HeLa cells (Table S1) …………………………S16

Flow cytometric analysis of calreticulin exposure in HeLa cells (Figure S19) …………………………S16

Flow cytometry density plots showing the phagocytosis (Figure S20) ……………………………………S17

References …… S17

Experimental

Materials. Reagents were used as received unless otherwise specified. All solvents used were of analytical grade or higher. Potassium tetrachloroplatinate (K_2PtCl_4) was purchased from Precious Metals Online. Acetone, acetic acid, acetonitrile, dichloromethane (DCM), and diethyl ether were obtained from Sigma-Aldrich. Hydrogen peroxide was obtained from VWR. Methanol was obtained from Honeywell. Deuterated solvents dd_6-dimethylsulphoxide (99.9%) and deuterium oxide (99.9%) were purchased from Cambridge Isotope Laboratories.

Synthesis of 2-aminoethyldiclofenacamide (enDCF). 2-(2,6-Dichloroanilino)-phenylacetic acid (DCF) was dissolved in a minimal amount of chloroform before 2 equivalents of 1,2-ethylenediamine (en) were added at room temperature. The solution was left to sit overnight, after which it produced large clear crystals, which were filtered and washed with chloroform. Yield 95%.^{1}H NMR (400 MHz, d-DMSO) δ 7.37 (d, 2H: CH, *J*= 8.34 Hz), 7.20 (d, 1H: CH, *J*= 7.14 Hz), 7.00 (m, 2H; CH), 6.82 (t, 1H, CH, *J*= 7.74 Hz), 6.37(d, 1H: CH, *J*= 8.19 Hz), 0.92 (m, H; NH), 4.1(m, H; NH).

*Synthesis of **1**; [Pt^{II}(5,6-dimethyl-1,10phenanthroline)(1S,2S-diaminocyclohexane)]$^{2+}$.* The synthesis of [Pt(56MePhen)(*SS*-dach)]$^{2+}$ (Pt^{II}56MeSS) was achieved using the published method.[1] [Pt^{II}(*SS*-dach)Cl_2] (307.5 mg; 85.4 mmol: 1 equiv.) and 56MePhen (164.5 mg: 91.3 mmol: 1.1 equiv.) were refluxed for 24 h, resulting in the solution that transforms from an opaque pale yellow to a clear yellow-orange solution. The reaction mixture volume was then reduced to allow purification *via* a Vac 20cc (5 g) C_{18} Sep-Pak© column connected to a pump apparatus with a UV detector (Bio-Rad, EM-1 Econo™ UV Monitor). The column was activated with methanol (20 mL) and then flushed with water (~40 mL) until the UV absorbance was equilibrated. The purified solution was then reduced under vacuum and freeze-dried. Yield 393.6 mg, 86.5%. ^{1}H NMR (400 MHz, D_2O) δ 8.78 (d, 1H: CH, *J*= 5.32Hz), 7.94 (dd, 1H: CH, *J*=8.48, 8.37 Hz), 8.90 (d, 1H: CH, *J*= 8.76 Hz), 2.64 (s, 6H; CH_3), 2.70 (m, 2H; CH_2), 2.21 (d, 2H; CH_2 *J*= 12.57 Hz), 1.65 (m, 2H; CH_2), 1.46 (d, 2H; CH_2 *J*= 12.49 Hz), 1.23 (m, 2H; CH_2), ^{1}H/^{195}Pt 9.66/-2759.2

*Synthesis of **2**; [Pt^{IV}(5,6-dimethyl-1,10-phenanthroline)(1S,2S-diaminocyclohexane)(Cl)$_2$]$^{2+}$.* The synthesis of [Pt^{II}(56MePhen)(*SS*-Dach)Cl_2]$^{2+}$ (Pt^{II}56MESS) was achieved using previously published methods.[2] The Pt^{II}56MESS(1 equiv) and *N*-chlorosuccinimide (2.4 equiv) were combined and left to react in a 9 mL solution of 1:1:1H_2O: EtOH: 1 M HCl. After approximately 2 h, the reaction was stopped by evaporation under a vacuum. To purify the product, the reaction was redissolved in water and eluted through a Vac 2Occ (5 g) C_{18} Sep-Pak© column connected to a pump apparatus with a UV detector (Bio-Rad, EM-1 Econo™ UV Monitor). The column was activated with methanol (20 mL) and then flushed with water (~40 mL) until the UV absorbance was equilibrated. The first and last bands of three to elute were identified as impurities, whilst the second band contained the major product. The complex was further purified using toluene in a soxhlet apparatus. If impurities remained after this process, the sample was further purified using the Reveleris® X2 flash chromatography system fitted with a Reveleris® reverse phase C_{18} 4 g column and eluted with MeOH and H_2O. Yield 34.7%.^{1}H NMR (400 MHz, D_2O) 9.14 (d, 1H: CH, J= 8.42 Hz), 8.24 (dd, 1H: CH, J=5.48, 8.56 Hz), 9.02 (d, 1H: CH, J= 5.42Hz), 2.77 (s, 6H; CH_3), 3.33 (m, 2H; CH_2), 2.36 (d, 2H; CH_2 J= 12.57 Hz), 1.72 (m, 2H; CH_2), 1.66 (d, 2H; CH_2 *J*= 10.71Hz), 1.30 (m, 2H; CH_2), ^{1}H/^{195}Pt 9.15/-653.5.

Synthesis of [Pt^{IV}(5,6-dimethyl-1,10-phenanthroline)(1S,2S-diaminocyclohexane)(DMSO)$_2$]$^{2+}$. Pt^{IV}PHENSSCl_2 (50 mg) was dissolved in 10 mL of DMSO and stirred at 40 °C overnight, at which point the suspension had fully dissolved. The crude product was then either used for further synthesis or extracted using 6 washes of 25 mL chloroform. Yield 96%.^{1}H NMR (400 MHz, d-DMSO) δ 11.85 (d, 2H: CH, J=5.40 Hz), 11.58 (d, 2H: CH, J= 8.55), 10.79 (m, 4H: CH/ CH_3), 5.30 (m, 2H: CH), 4.72 (m,12H: CH_3), 4.57 (m, 2H: CH), 3.96 (m, 2H: CH), 3.83 (m, 2H: CH), 3.53 (m, 2H: CH), ^{195}Pt -600.8 ppm.

Synthesis of ***3****; [Pt^{IV}(5,6-dimethyl-1,10-phenanthroline)(1S,2S-diaminocyclohexane)(Cl)(2-aminoethyldiclofenacamide)]$^{3+}$*. Pt^{IV}56MESS(DMSO)$_2$ solution containing 100 mg, 1 equiv and 1.1 equivalents of 2-aminoethyldiclofenacamide (DCF-en) were stirred for 20 min- the solution was initially bright orange and upon color change to dark purple 20 mL water was added and the reaction was stirred at 70 °C for and additional 20 min .The volume was reduced and purified using the flash chromatography unit using a 10-100% water to MeOH gradient. The fraction containing the product was dried before being taken up in ~5 mL of water and freeze dried to produce a pale-yellow powder. Yield 85%, ^{1}H NMR (400 MHz,d-Acetone) δ (56MEENSS peaks) 8.89 (d, 2H: CH, J= 8.42Hz), 8.01 (dd, 2H: CH, J= 8.13, 8.41Hz), 9.11 (d, 2H: CH, J= 5.26 Hz), 8.13 (s,2H; CH), 2.69, (m, 2H; CH_2), 2.25 (d, 2H; CH_2, J=12.81Hz), 1.69 (m, 2H; CH_2), 1.54 (m, 2H; CH_2), 1.29 (m, 2H; CH_2): (en-DCF peaks) 7.23 (d, 2H: CH, J= 8.08 Hz), 7.12(d, 2H: CH, J= 7.50 Hz), 6.91(t, 4 H: CH, J= 8.03 Hz), 6.85(t, 4 H: CH, J= 7.72 Hz), 6.70(t, 2H: CH, J= 7.54 Hz),6.21(d, 4 H: CH, J= 7.95 Hz), HPLC elution 4.06 min at a 0-100% H_2O-ACN slope, 97.02% purity.

Synthesis of ***4****; [Pt^{IV}(5,6-dimethyl-1,10-phenanthroline)(1S,2S-diaminocyclohexane)(2-aminoethyldiclofenacamide)$_2$]$^{4+}$*. Pt^{IV}56MESS(DMSO)$_2$ (100 mg, 1 equiv) and 2.2 equivalents of 2-aminoethyldiclofenacamide (DCF-en) were dissolved in 30 mL water and stirred at 70 °C for 20 min before being taken to dryness under vacuum, producing a pale yellow residue. Then 50 mL of absolute ethanol was added to the flask and let to stir at 40 °C for a further 12 h. The resulting grey suspension was filtered, and the filtrate reduced under pressure to ~3 mL. The crude product was precipitated with 20 mL diethyl ether. The precipitate was filtered and washed thrice with ether (3 mL). To remove all traces of solvent, the produce was taken up in ~5 mL of water and freeze-dried to produce a pale-yellow powder. Yield 85%, ^{1}H NMR (400 MHz, d-Acetone) δ (56MESS peaks) 8.89 (d, 2H: CH, J= 8.42Hz), 8.01 (dd, 2H: CH, J= 8.13, 8.41Hz), 9.11 (d, 2H: CH, J= 5.26 Hz), 8.13 (s,2H; CH), 2.69, (m, 2H; CH_2), 2.25 (d, 2H; CH_2, J=12.81Hz), 1.69 (m, 2H; CH_2), 1.54 (m, 2H; CH_2), 1.29 (m, 2H; CH_2): (en-DCF peaks) 7.23 (d, 2H: CH, J= 8.08 Hz), 7.12(d, 2H: CH, J= 7.50 Hz), 6.91(t, 4 H: CH, J= 8.03 Hz), 6.85(t, 4 H: CH, J= 7.72 Hz), 6.70(t, 2H: CH, J= 7.54 Hz),6.21(d, 4 H: CH, J= 7.95 Hz). HPLC elution 9.98 min at a 0-100% H_2O-ACN slope, 97.73% purity.

Detailed description of synthesis

The intermediate, Pt^{IV}56MeSSCl$_2$ (complex **2**; Figure 1), was isolated utilizing previously published methods, using *N*-chloro succinimide to oxidize Pt(II) compound **1**.[2] Further purification was not required because, upon precipitation, the succinimide by-product was separated from the dichloride intermediate, and the resulting solution was instead dried under vacuum. DMSO was added to the dried Pt(IV) complex **2.**The synthetic precursor Pt^{IV}56MESS(DMSO)$_2$ was used because DMSO is a good leaving group that allows efficient coordination of en-DCF into the axial positions. Based on a modified, published method,[3] DMSO was added to a stirring solution of **2** at a low temperature to produce Pt^{IV}56MeSS(DMSO)$_2$. The reaction can be easily monitored as the starting reagent is not soluble in DMSO, so a clear solution indicates that the chlorido ligands have exchanged with the solvent. Optimization of the reaction conditions revealed that for every 100 mg of Pt(IV) complex **2**, a minimum of 7 mL of DMSO is required for chloroform extraction to successfully separate Pt^{IV}56MeSS(DMSO)$_2$ from the reaction solution containing DMSO. In ^{1}H and ^{195}Pt NMR, all the resonances could be assigned. The amine peaks appear much further upfield than expected, resonating close to the phenanthroline peaks. The resulting solution had no trace of Pt(II) complex **1** nor the Pt(IV) complex **2** intermediate. The DMSO solution was used for the following reactions where 5 equiv. of en-DCF is added directly into the crude Pt^{IV}56MESS(DMSO)$_2$ solution, at which point the color changed from pale yellow to dark orange and back to almost clear before becoming dark green, this all occurred within the space of a minute; after several minutes the solution became purple black in color. The reaction was stirred at

70 °C for at least 20 min, but this time was increased in instances when the en-DCF crystals did not fully dissolve, and this had no noticeable impact on the purity or quantity of the yield. At this point, the monosubstituted product could be collected *via* flash chromatography, or the DMSO reaction solution could be diluted by a factor of 4 with absolute ethanol and subsequently stirred overnight at 40 °C to produce the disubstituted product. The ethanol was removed *via* evaporation, and the remaining liquid was filtered before being eluted through the flash chromatography system using a C_{18} reverse-phase column with a low water-to-methanol gradient. Two peaks eluted; however, only one contained the final product along with some impurities and the other contained nothing recognizable in a proton NMR. This final product fraction was reduced to a minimal volume and rerun through the same column but at a higher methanol gradient to remove impurities. The isolated product was an off-white powder that was slightly soluble in water but very soluble in methanol. The typical yield from this reaction is 85% with respect to Pt(II) complex **1**.

Pt^{II}56MESS (complex 1) ^{195}Pt NMR spectra

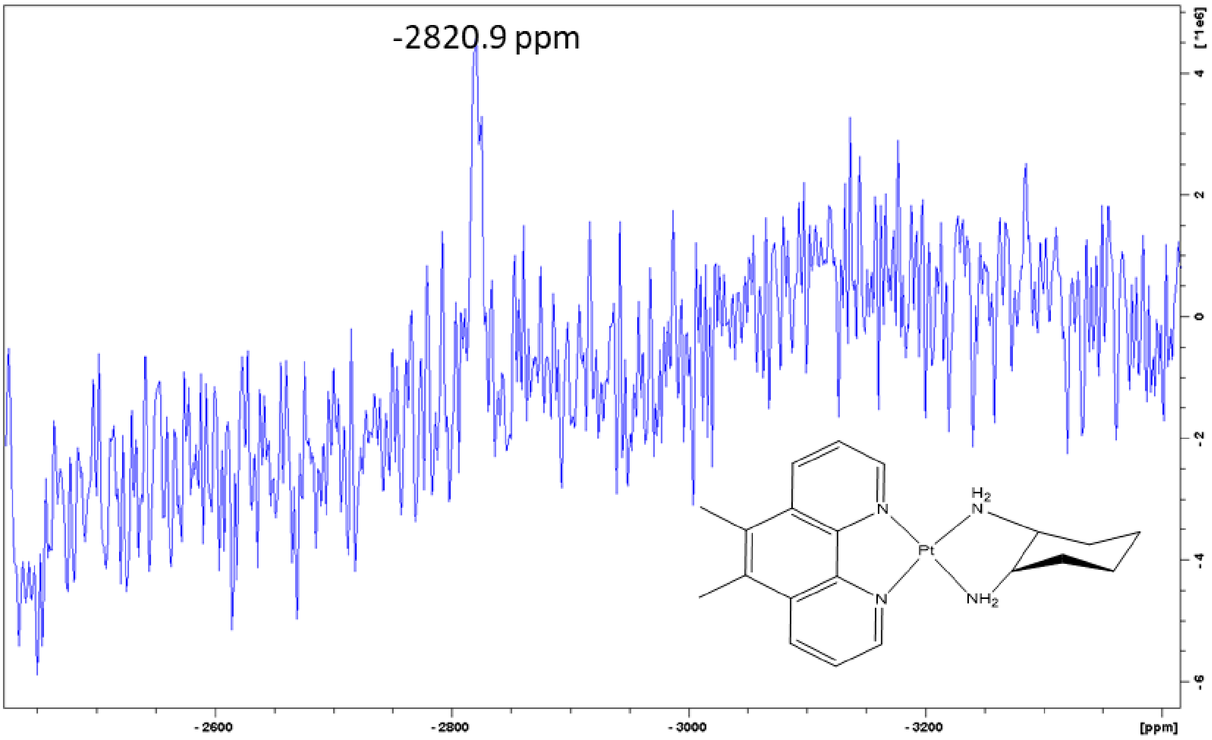

Figure S1: The ^{195}Pt spectrum of complex **1** in D_2O, performed on a Bruker Avance 400 MHz NMR spectrometer.

en-DCF ^{1}H NMR spectra

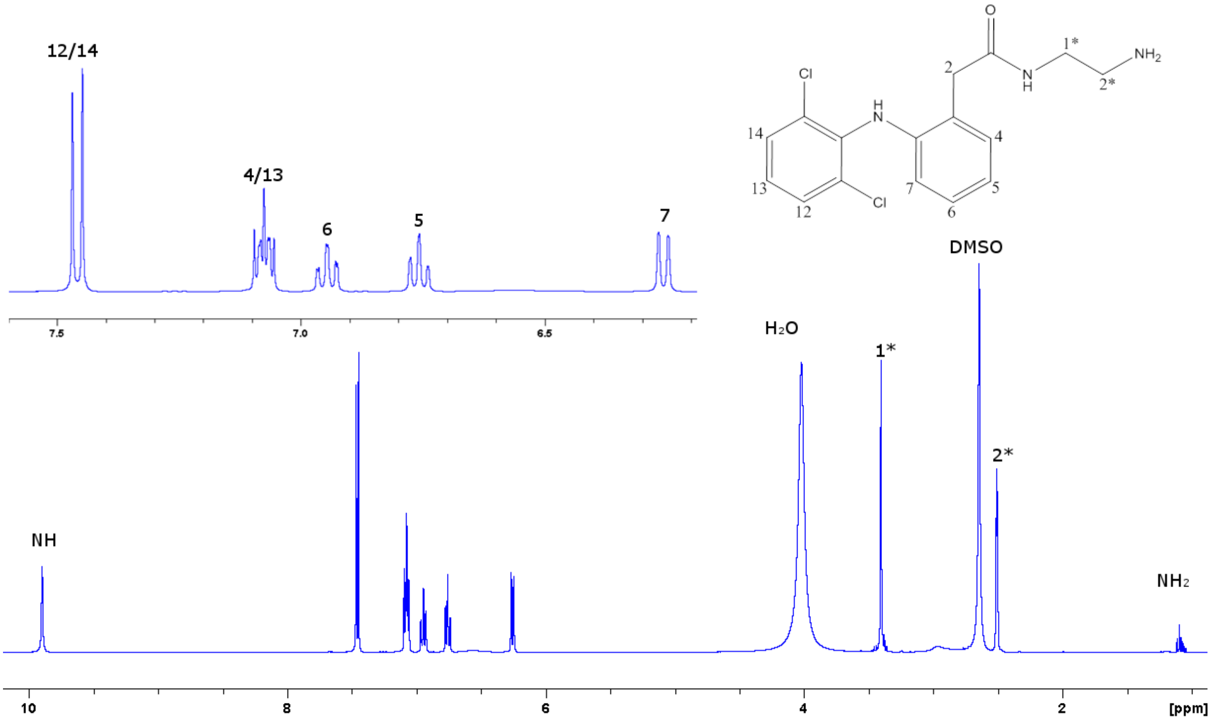

Figure S2: The ^{1}H spectrum of en-DCF in D_2O, performed on a Bruker Avance 400 MHz NMR spectrometer.

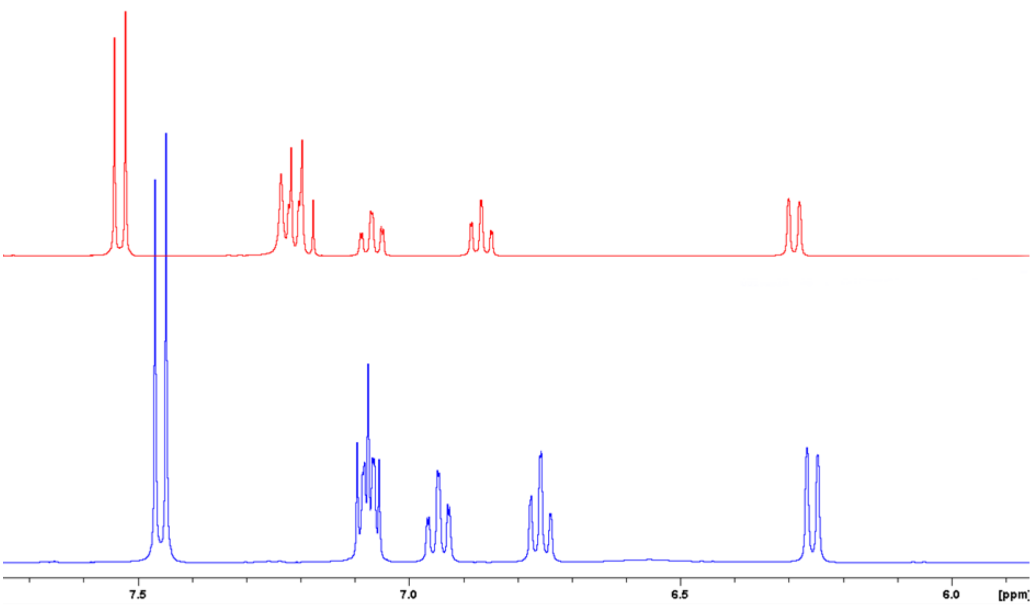

Figure S3: The ^{1}H NMR spectra of en-DCF in DMSO (blue) in comparison to the ^{1}H NMR spectra of DCF in DMSO (red); performed on a Bruker Avance 400 MHz NMR spectrometer.

PHENSS(DMSO)$_2$ (in DMSO) ^{1}H NMR spectra

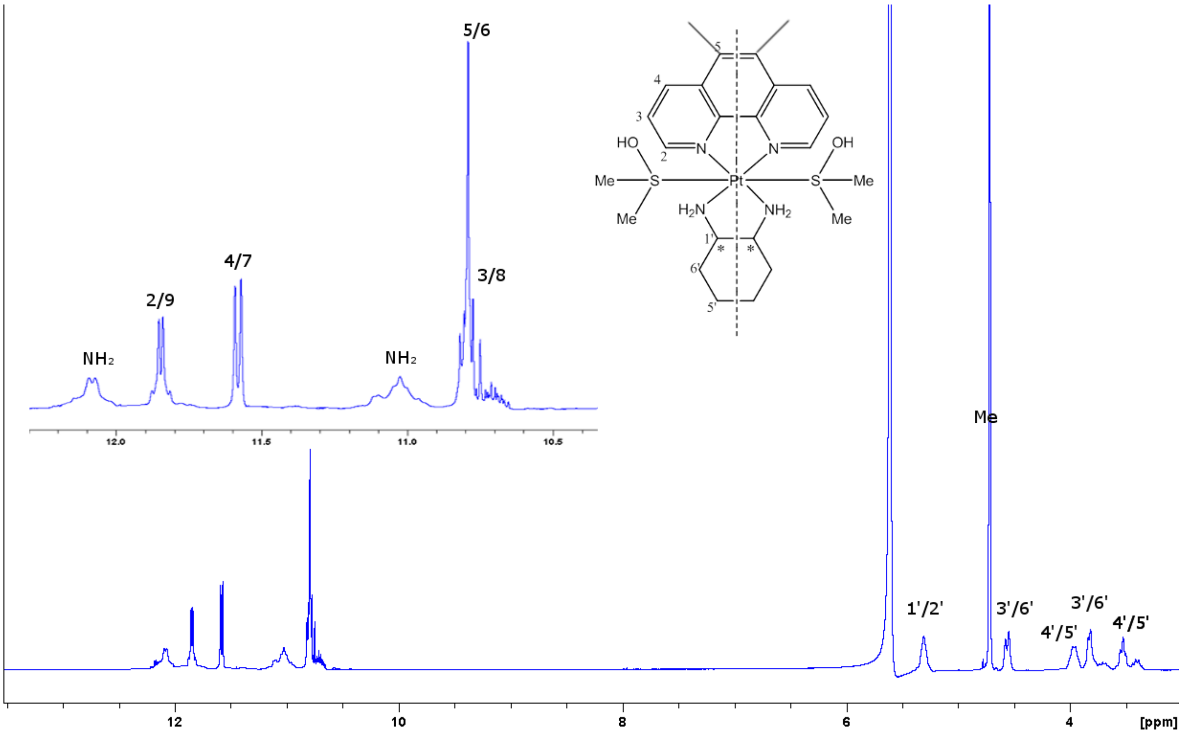

Figure S4: The ^{1}H spectrum of Pt^{IV}56MESS(DMSO)$_2$ in d-DMSO, performed on a Bruker Avance 400 MHz NMR spectrometer. Insert: an expansion of 12.4-10.4 ppm region.

Pt^{IV}56MESS(DMSO)$_2$ (in DMSO) ^{195}Pt NMR spectra

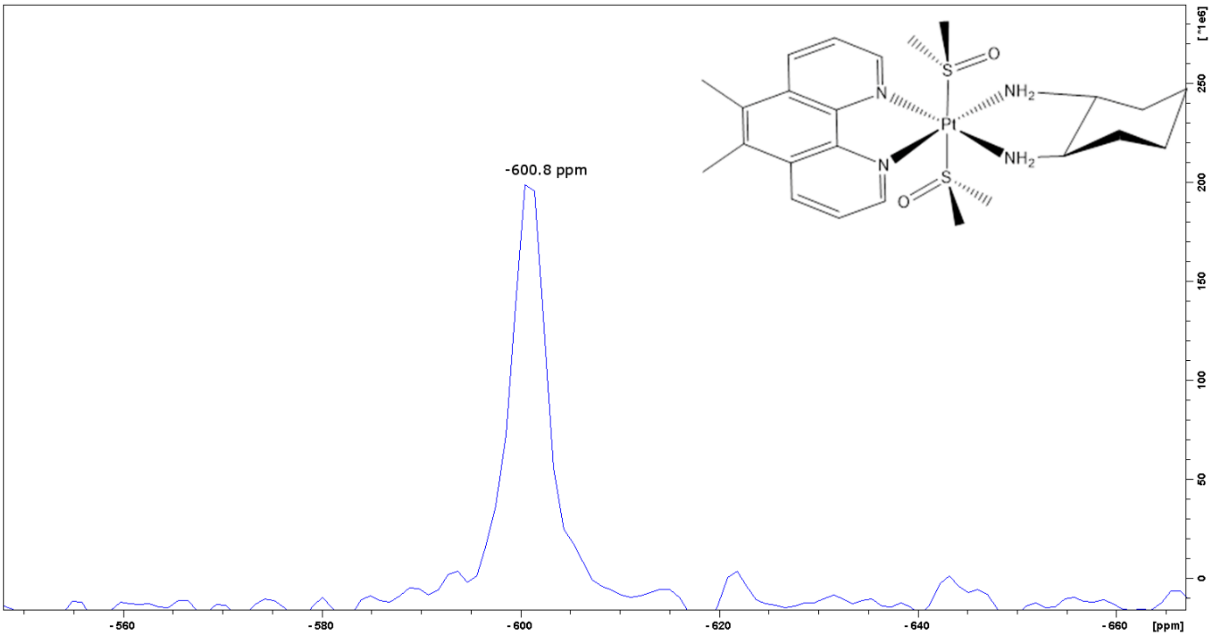

Figure S5: The ^{195}Pt spectrum of Pt^{IV}56MESS(DMSO)$_2$ in d-DMSO, performed on a Bruker Avance 400 MHz NMR spectrometer.

Pt^{IV}56MESSenDCF$_2$ ^{1}H NMR spectra

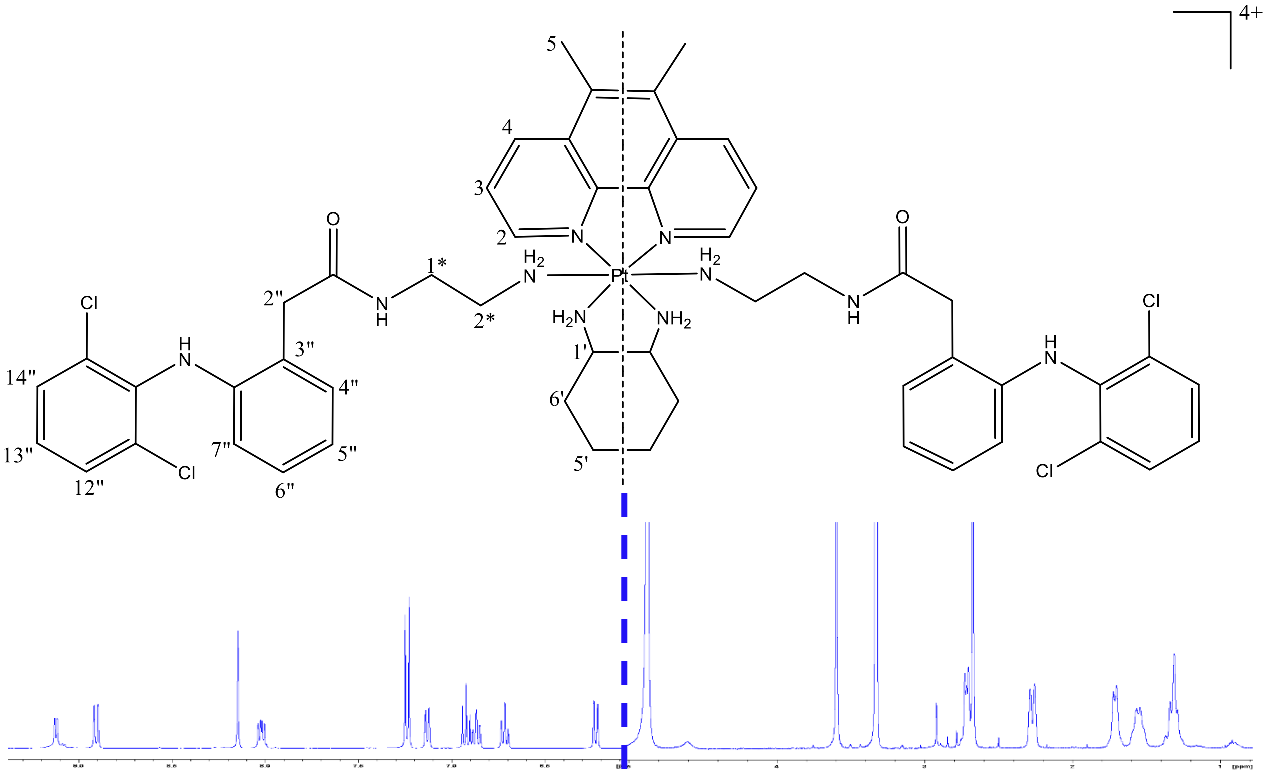

Figure S6: The ^{1}H spectrum of complex **4** in D_2O, performed on a Bruker Avance 400 MHz NMR spectrometer.

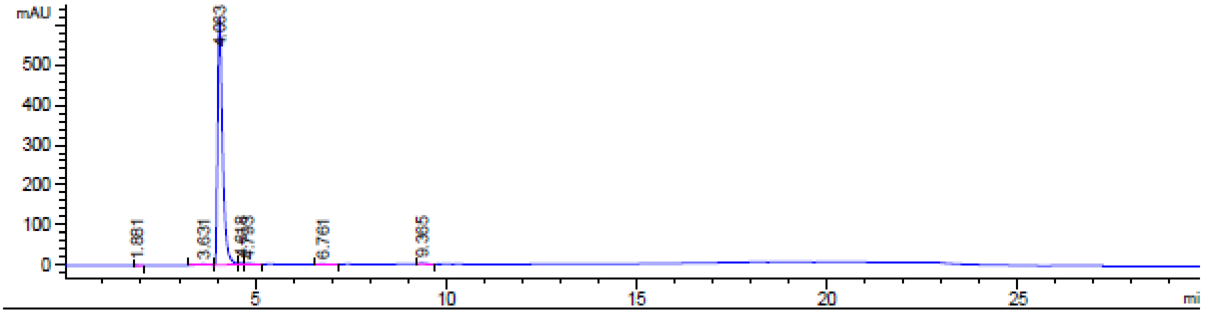

Figure S7: The HPLC trace of Pt^{IV}56MESS(OH)(DCF), measured on an Agilent Technologies 1260 Infinity machine equipped with a Phenomenex Onyx™ Monolithic C18 reverse phase column (100 × 4.6 mm, 130 Å). Sample solutions were made up in H_2O and injected at a 0-100 gradient H_2O to ACN over 15 minutes with a 15-minute flush in-between samples.

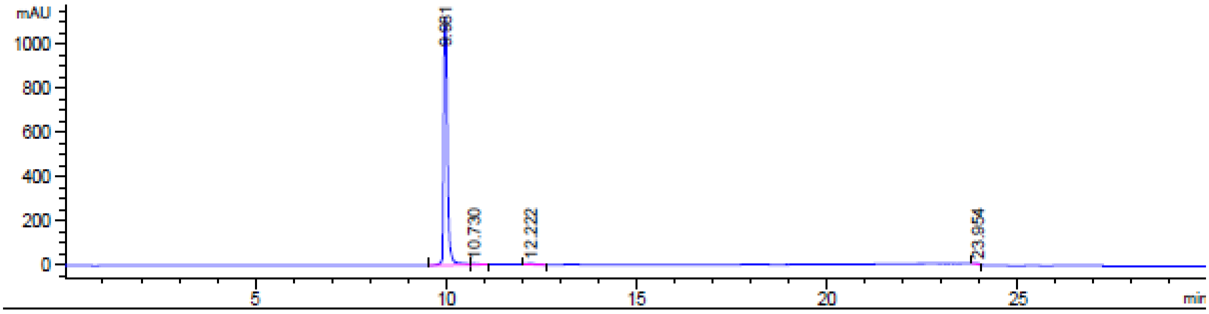

Figure S8: The HPLC trace of complex **4** measured on an Agilent Technologies 1260 Infinity machine equipped with a Phenomenex Onyx™ Monolithic C18 reverse phase column (100 × 4.6 mm, 130 Å). Sample solutions were made up in H_2O and injected at a 0-100 gradient H_2O to ACN over 15 min with a 15-min flush in-between samples.

Reduction studies (Figures S9-16), were undertaken whereby the reducing agent was added in excess and the reduction monitored by NMR. This study was undertaken with 3 and 10 equivalents of ascorbic acid and 4 and 10 equivalents of GSH. All had long reduction half-lives and in the case of 10 equivalents of GSH, after 3.5 weeks we were forced to end the experiment as the deuterated solvent had degraded to the point a clean spectra could not be obtained.

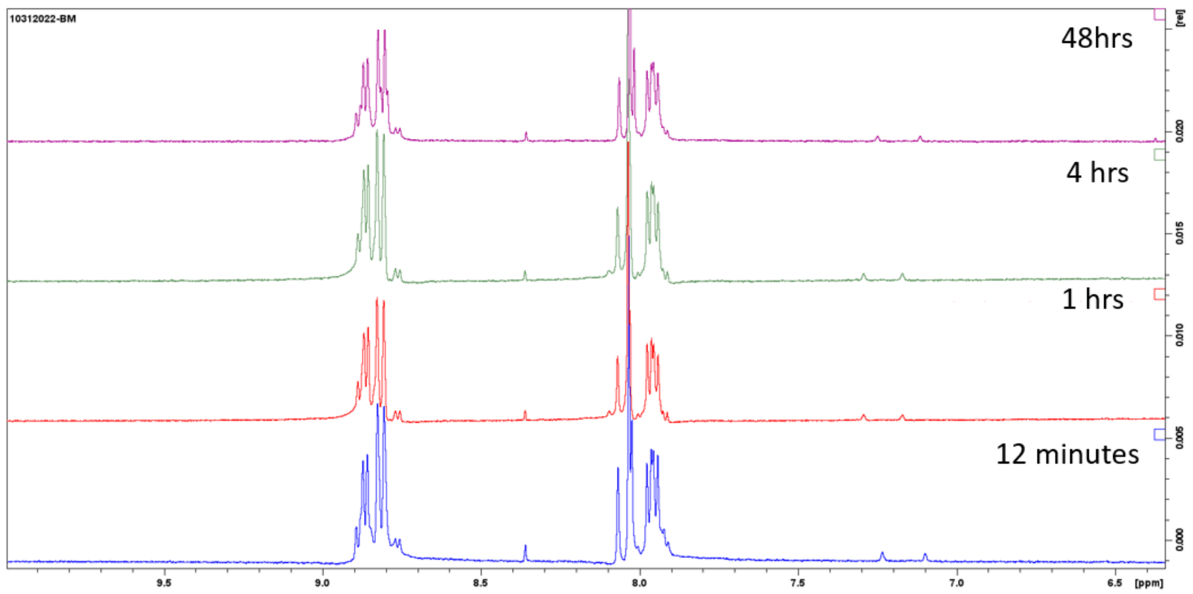

Figure S9: The ^{1}H spectra of complex **4** in D_2O PBS solution, 12 min (blue), 1 h (red) 4 h (green) and 48 h (purple) after the addition of 3 equivalents of ascorbic acid, performed on a Bruker Avance 400 MHz NMR spectrometer at 37 °C.

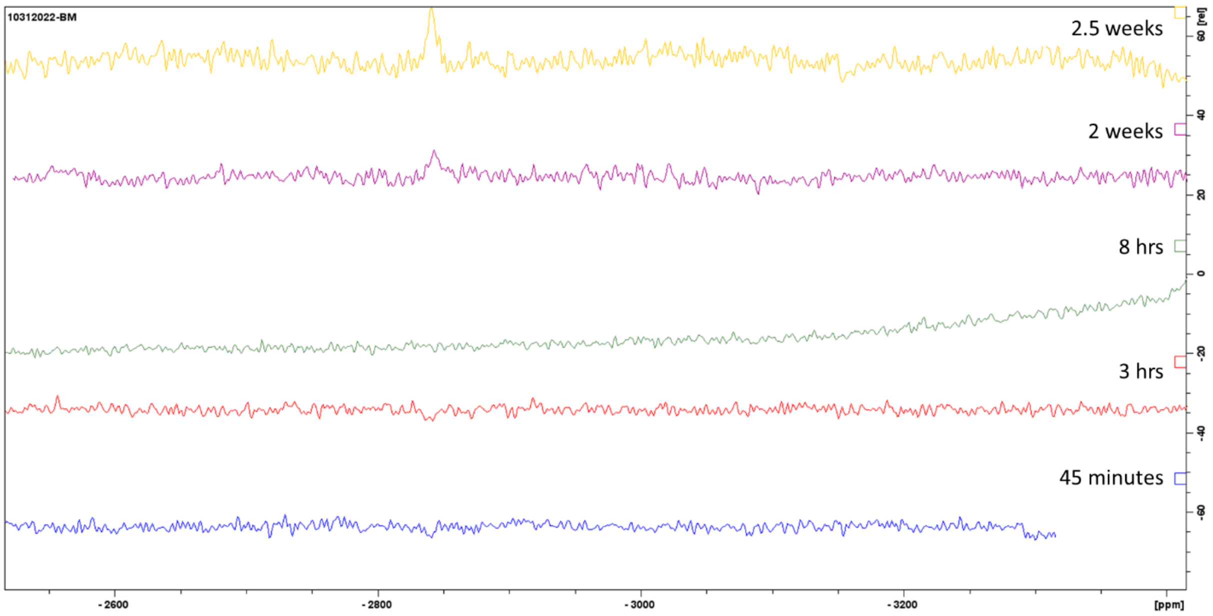

Figure S10: The ^{195}Pt spectra of complex **4** in D_2O PBS solution, 45 min (blue), 3 h (red) 8 h (green), 2 weeks (purple) and 2.5 weeks (yellow) after the addition of 3 equivalents of ascorbic acid, performed on a Bruker Avance 400 MHz NMR spectrometer at 37 °C for the first 8 h both the 2 and 2.5 weeks scans done at 298 K.

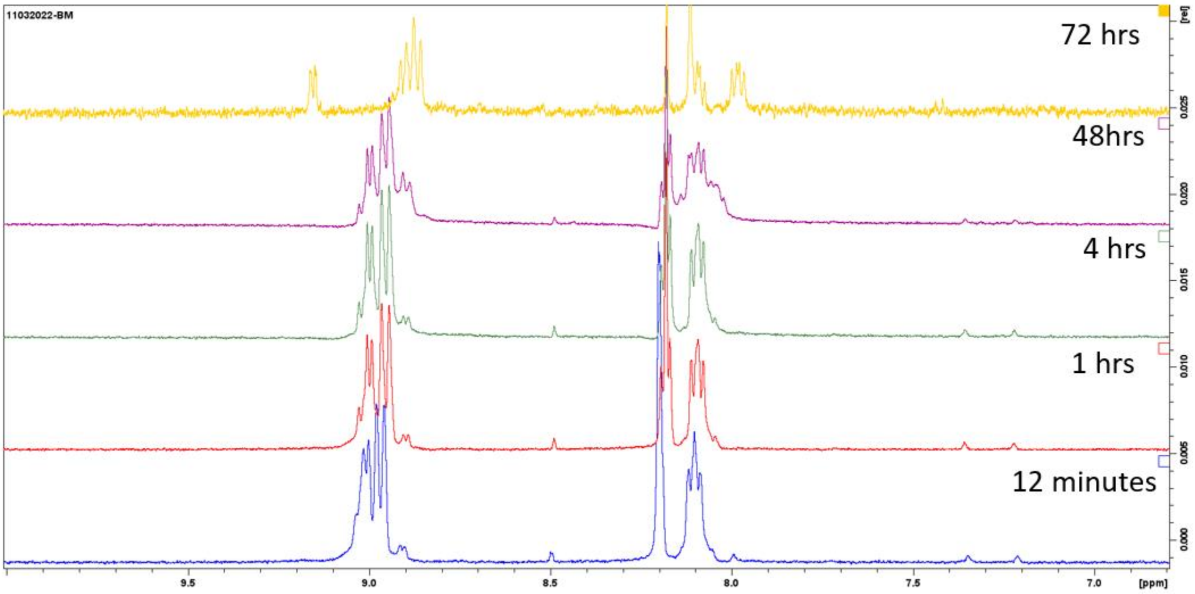

Figure S11: The ^{1}H spectra of complex **4** in D_2O PBS solution, 12 min (blue), 1 h (red), 4 h (green), 48 h (purple), and 72 h (yellow) after the addition of 4 equivalents of GHS, performed on a Bruker Avance 400 MHz NMR spectrometer at 37 °C.

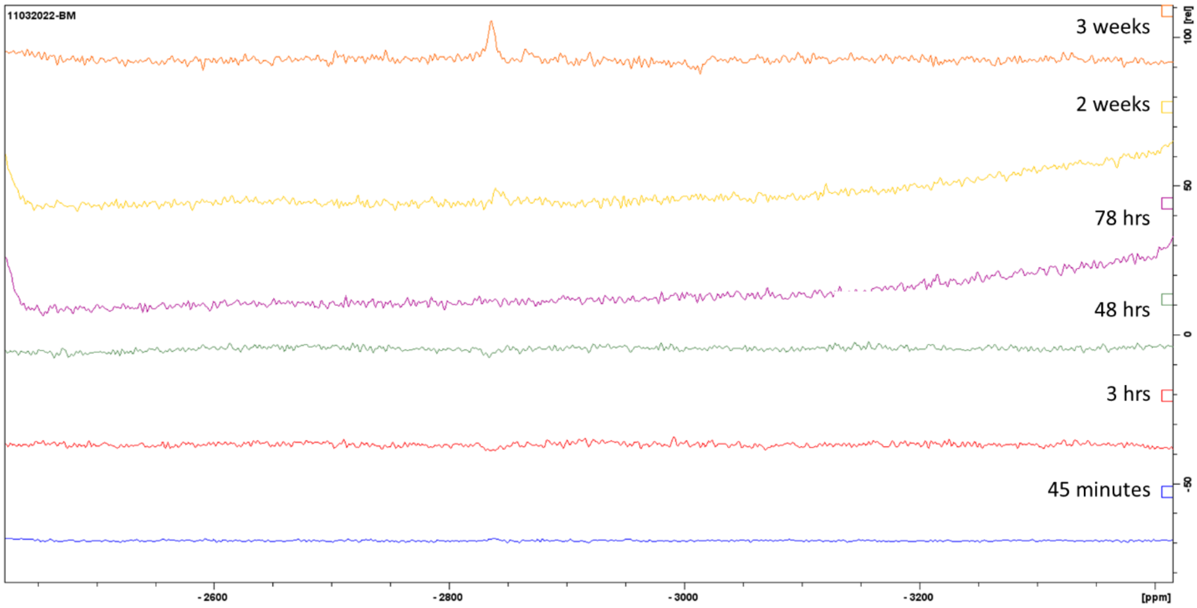

Figure S12: The ^{195}Pt spectra of complex **4** in D_2O PBS solution, 45 min (blue), 3 hr (red) 48 h (green), 78 h (purple), 2 weeks (yellow) and weeks (orange) after the addition of 4 equivalents of GHS, performed on a Bruker Avance 400 MHz NMR spectrometer at 37° C for the first 8 h for the remainder 298 K.

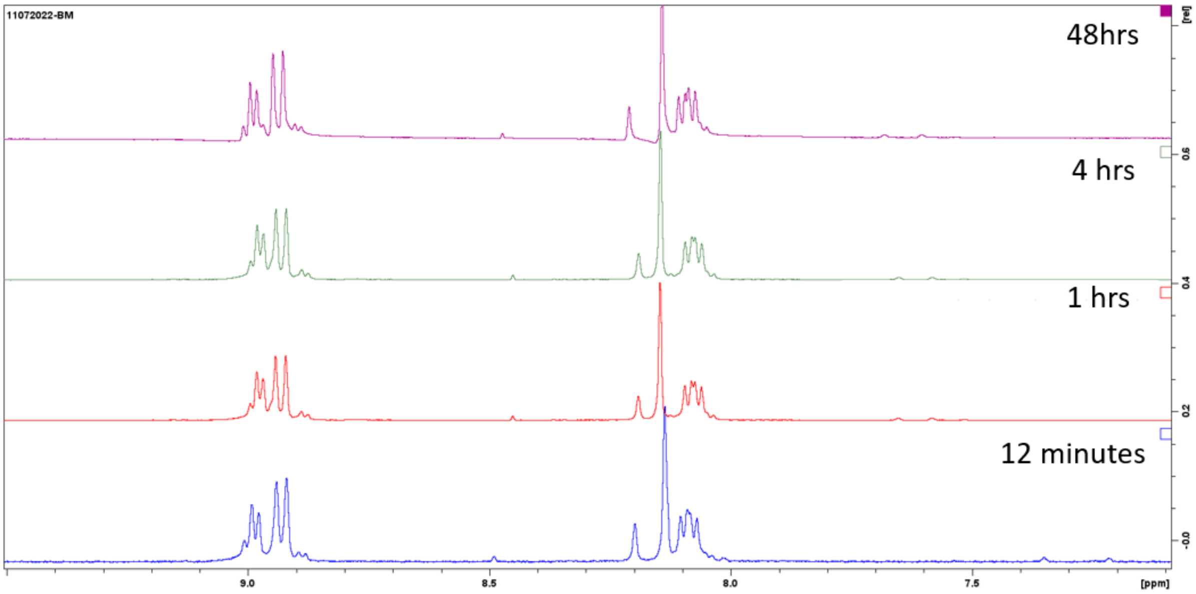

Figure S13: The ^{1}H spectra of complex **4** in D_2O PBS solution, 12 min (blue), 1 h (red) 4 h (green) and 48 h (purple) after the addition of 10 equivalents of ascorbic acid , performed on a Bruker Avance 400 MHz NMR spectrometer at 37 °C

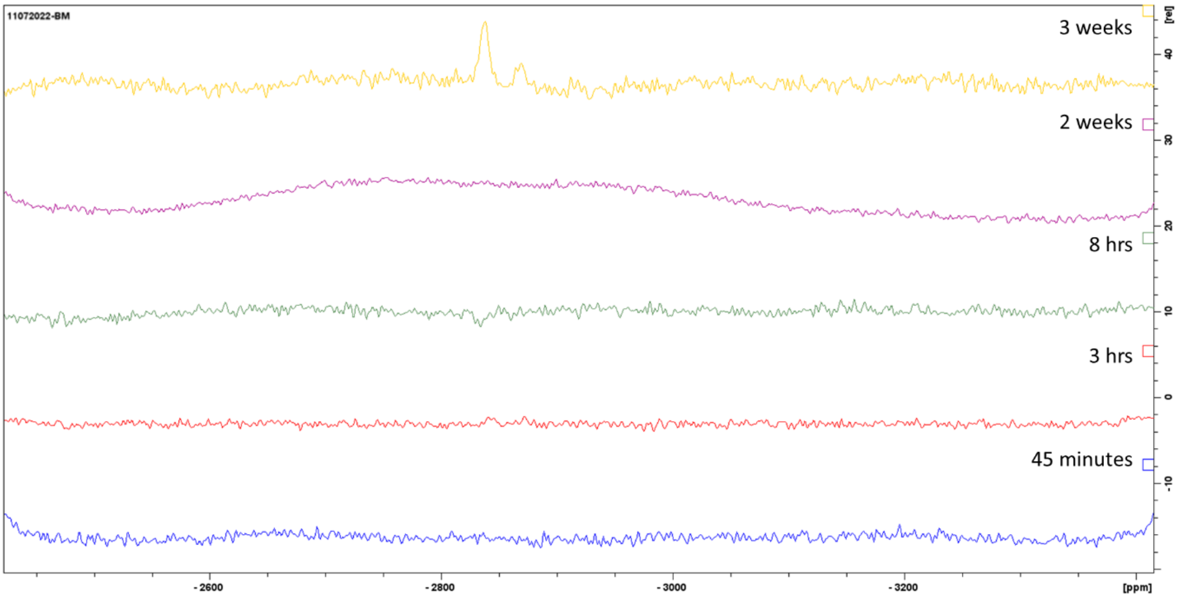

Figure S14: The ^{195}Pt spectra of complex **4** in D_2O PBS solution, 45 min (blue), 3 h (red) 8 h (green), 2 weeks (purple) and 3 weeks (yellow) after the addition of 10 equivalents of ascorbic acid, performed on a Bruker Avance 400 MHz NMR spectrometer at 37 °C for the first 8 h for the remainder 298 K.

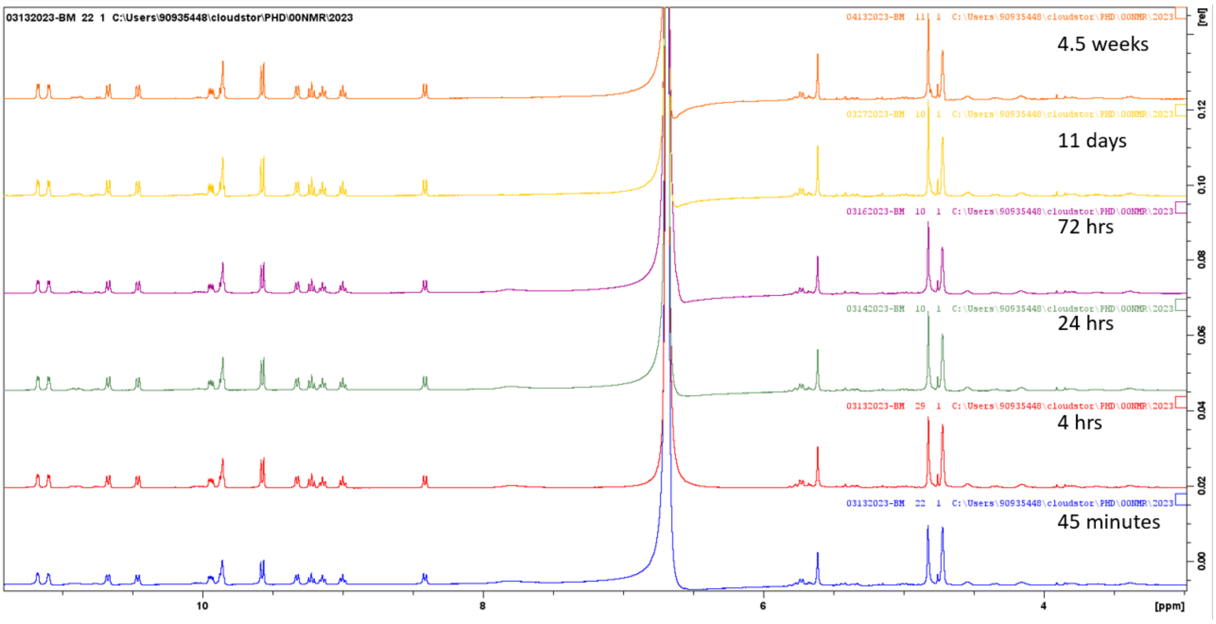

Figure S15: The ^{1}H spectra of complex **4** in D_2O PBS solution, 45 min (blue), 4 h (red) 24 h (green), 72 h weeks (purple), 11day (yellow) and, 4.5 weeks (orange) after the addition of 10 equivalents of GSH, performed on a Bruker Avance 400 MHz NMR spectrometer at 37 °C.

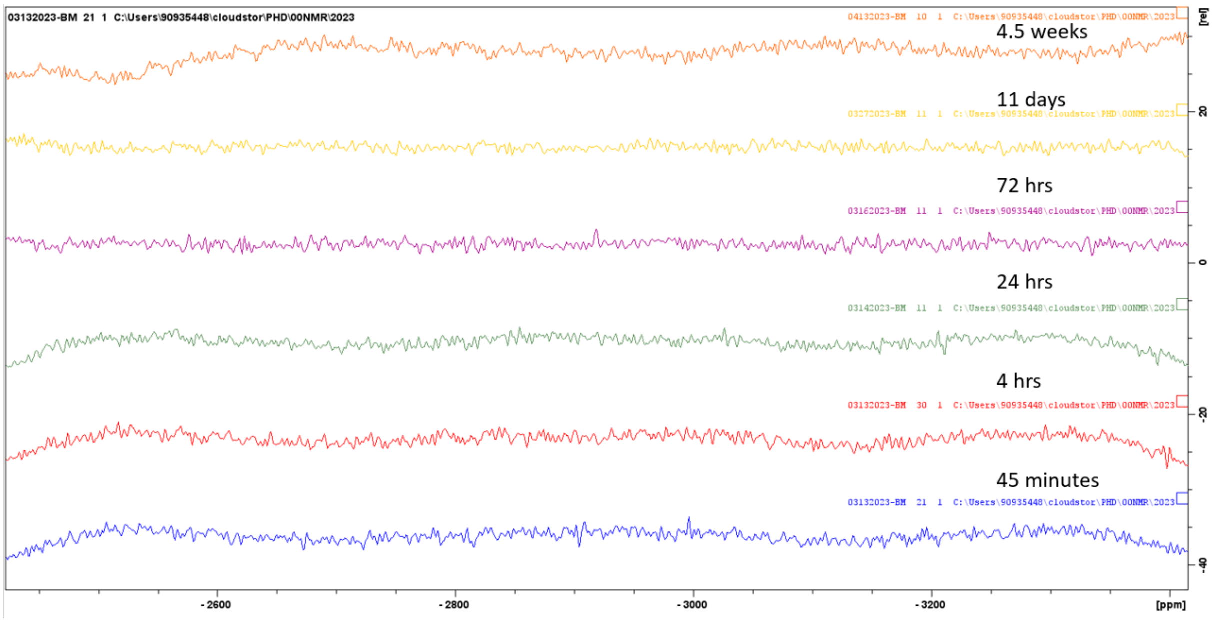

Figure S16: The ^{195}Pt spectra of complex **4** in D_2O PBS solution, 45 min (blue), 4 h (red) 24 h (green), 72 hrs weeks (purple), 11day (yellow) and, 4.5 weeks (orange) after the addition of 10 equivalents of GSH performed on a Bruker Avance 400 MHz NMR spectrometer at 37 °C for the first 8 h for the remainder 298 K.

Determination of the reduction potentials by cyclic voltammetry

We determined the cathodic reduction potentials for Pt(IV) complexes **3** and **4** with six nitrogen donors using cyclic voltammetry in conjunction with a glassy carbon electrode. The values of these potentials measured against Ag/AgCl 3M KCl reference electrode in PBS were for complexes **3**, and **4** -1.135 V, and -1.120 V, respectively. These values of the reduction potential were more negative than those determined for similar Pt(IV) complexes derived from complex **1** with only four nitrogen donors under similar conditions.[4] This suggests that complexes **3** and **4** might be less easily reducible. However, it must be noted that the experimental data already published revealed a low correlation between cytotoxicity and reduction rate.[5-8]

The effect of incubating complex 4 with HeLa cell extract

HPLC analysis showed a significant decrease in the peak corresponding to complex **4**, which was accompanied by the formation of a new product/s, whose retention time did not match that of free DCFen or DCF (Figure S17). This new peak was also observed after incubation of either DCF or enDCF with the cell extract, indicating that it is a product of DCF metabolism by enzymes present in the cell extract. Moreover, small peaks corresponding to reduced Pt(II) complex **1** and DCF were also seen on chromatogram after 25 min of incubation with extract, indicating that **4** can, at least in some extent, undergo reduction.

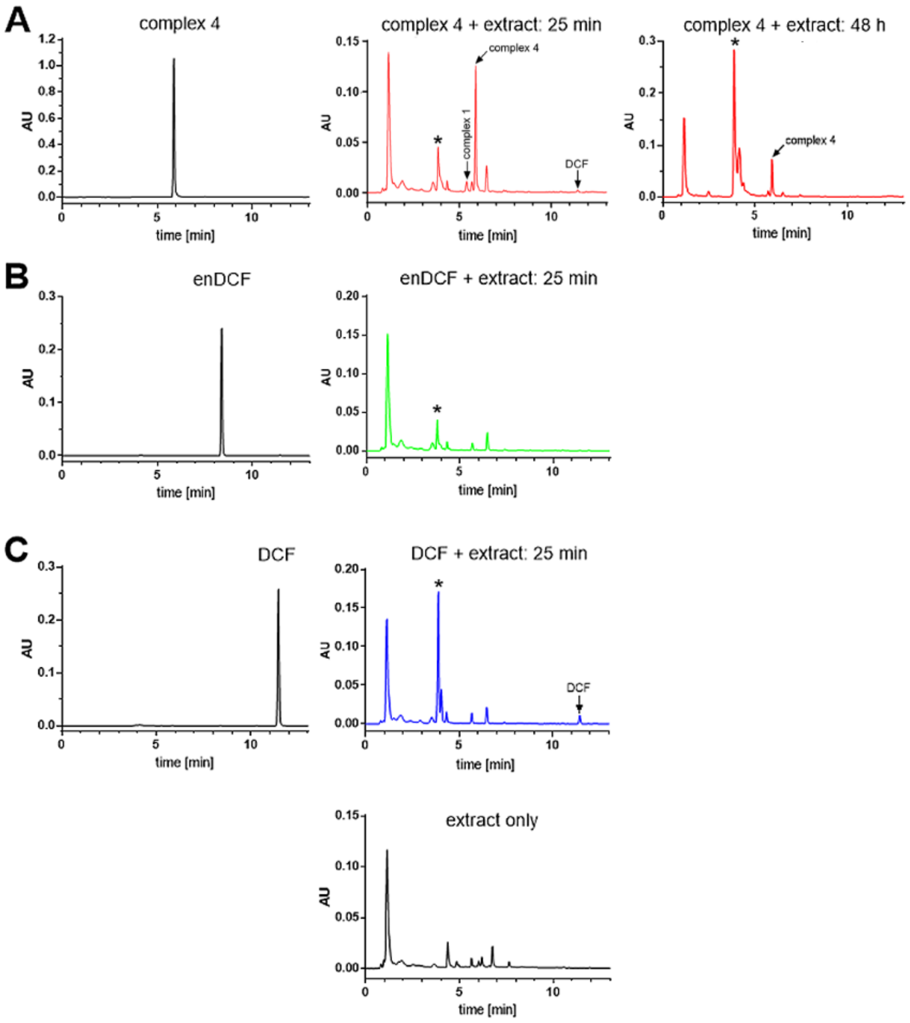

Figure S17: A representative chromatograms of **4** (A), enDCF (B) and DCF (C) either in buffer (left panels) or after incubation with high-molecular mass fraction (>3kDa) of HeLa cell extract for indicated time. The asterisk indicates a new product formed after incubation, which is apparently a metabolite of DCF. Note: For analytical reasons, it is necessary to remove excess of proteins from the sample before its application to the HPLC column. However, DCF and its metabolites bind effectively to the proteins,[9] as well as Pt(II) complex **1**.[10, 11] This limits the quantitative analysis of the chromatograms, so their evaluation is restricted to qualitative evaluation.

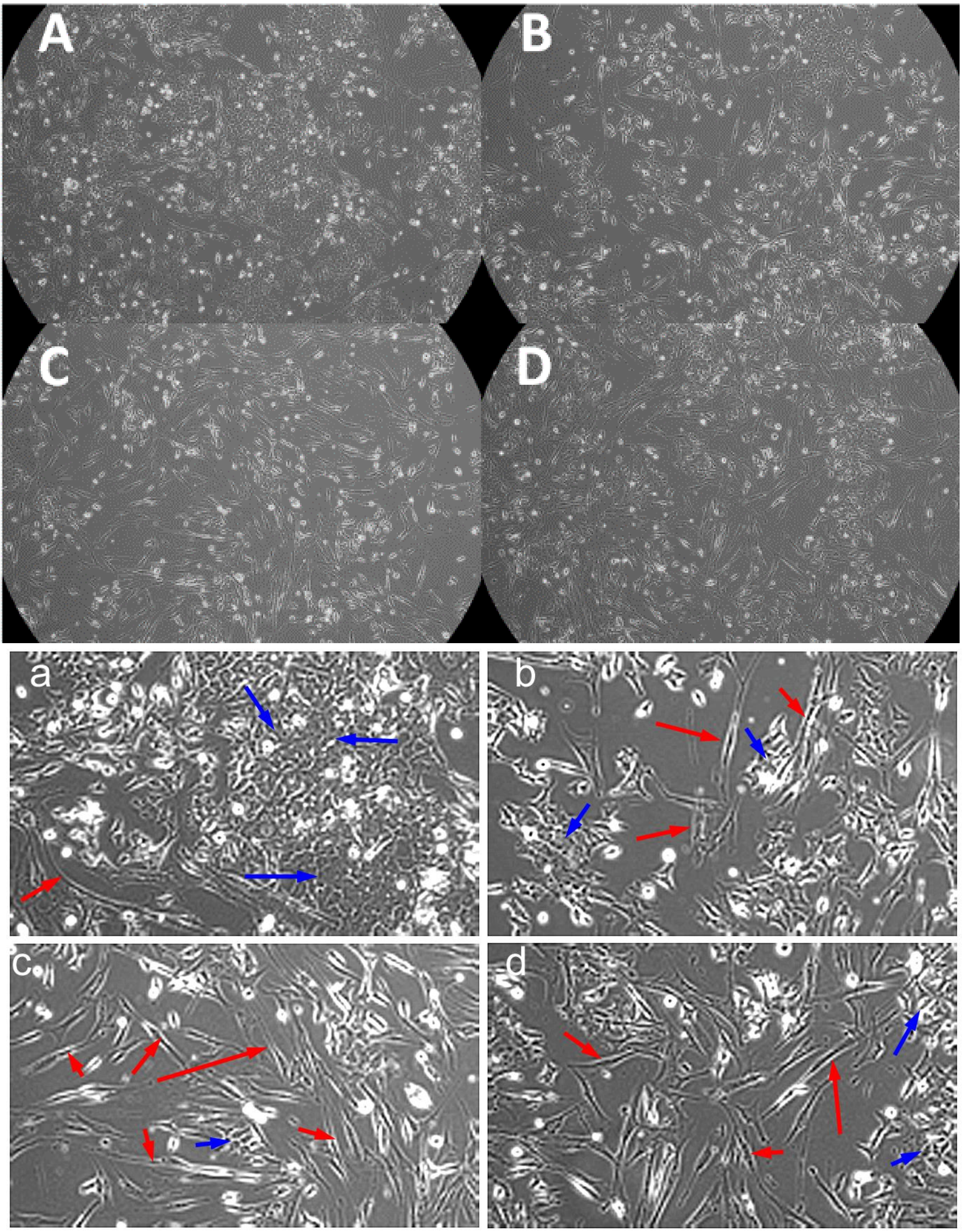

Figure S18: Co-cultured HCT-116 (round) and MRC-5 (spindle-like) cells after 72 h of treatment with complex **1** (panel B), complex **3** (panel C) or complex **4** (panel D). Control, untreated cells are shown in panel A. Panels a, b, c, and d show a section cropped from the center of dishes A, B, C, and D, respectively. Blue arrows point to representative HCT-116 cells and red arrows point to typical prolonged spindle-like MRC-5 cells.

Table S1: The amount of Pt associated with DNA isolated from HeLa cells treated with a 1 μM concentration of tested compounds for 6 h[a].

Complex	pg Pt/μg DNA
**1**	2.0 ± 0.2
**2**	1.9 ± 0.1
**3**	2.3 ± 0.2
**4**	4.6 ± 0.3
**cisplatin**	2.8 ± 0.2

[a]Data represent mean ± SD from at least three independent experiments.

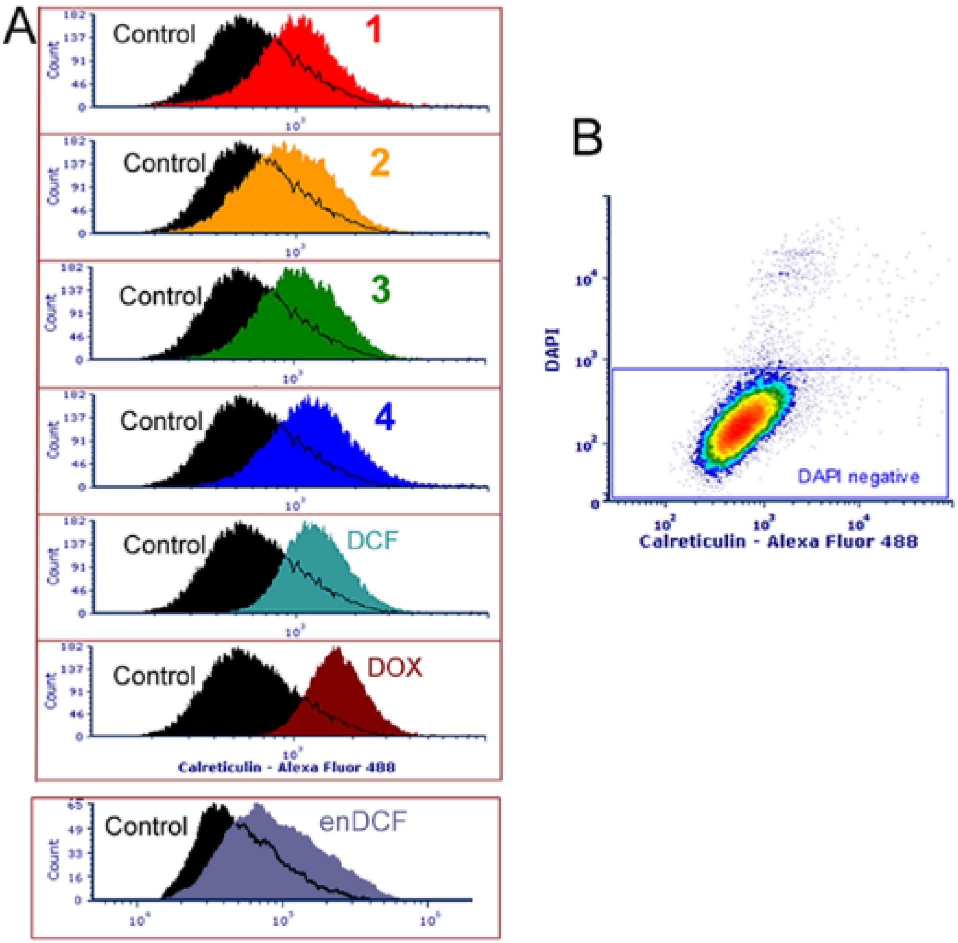

Figure S19: A. Representative histograms obtained by flow cytometric analysis of calreticulin exposure in HeLa cells treated with indicated compounds. B. An example of gating DAPI negative population. Only DAPI-negative cells were taken for the evaluation.

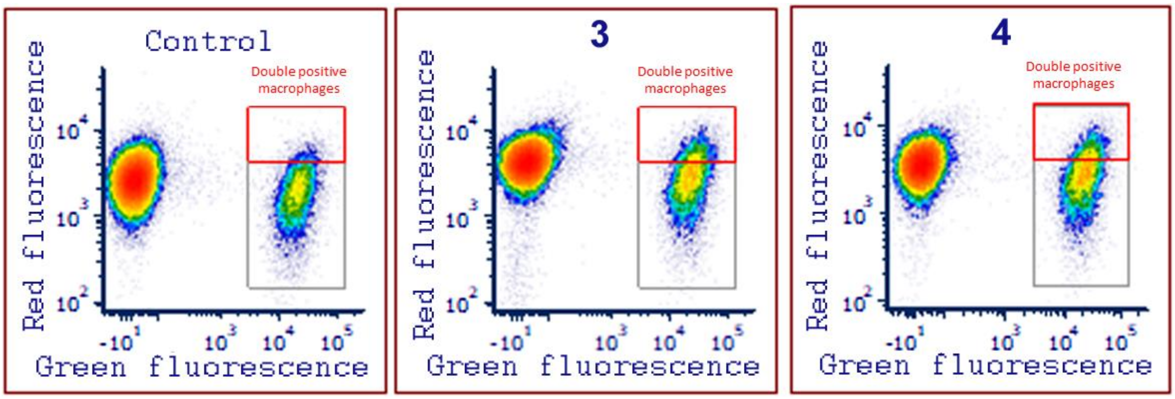

Figure S20: Representative flow cytometry density plots showing the phagocytosis of control, untreated cells (left panel) or after the cells were treated with complex **3** (middle panel) or complex **4** (right panel).

References

1. Wheate, N. J.; Taleb, R. I.; Krause-Heuer, A. M.; Cook, R. L.; Wang, S.; Higgins, V. J.; Aldrich-Wright, J. R. Novel platinum(II)-based anticancer complexes and molecular hosts as their drug delivery vehicles. *Dalton Trans.* **2007**, 5055 - 5064.
2. McGhie, B. S.; Sakoff, J.; Gilbert, J.; Aldrich-Wright, J. R. Synthesis and characterisation of platinum(IV) polypyridyl complexes with halide axial ligands. *Inorg. Chim. Acta* **2019,** 495, 118964.
3. Crosby, S. H.; Clarkson, G. J.; Deeth, R. J.; Rourke, J. P. Platinum(IV) DMSO complexes: Synthesis, isomerization, and agostic intermediates. *Organometallics* **2010,** 29, 1966-1976.
4. Harper, B. W. J.; Friedman-Ezra, A.; Sirota, R.; Petruzzella, E.; Aldrich-Wright, J. R.; Gibson, D. Probing the interactions of cytotoxic [Pt(1S,2S-DACH)(5,6-dimethyl-1,10-phenanthroline)] and its Pt^{IV} derivatives with human serum. *ChemMedChem* **2017,** 12, 510-519.
5. Hambley, T. W.; Battle, A. R.; Deacon, G. B.; Lawrenz, E. T.; Fallon, G. D.; Gatehouse, B. M.; Webster, L. K.; Rainone, S. Modifying the properties of platinum(IV) complexes in order to increase biological effectiveness. *J. Inorg. Biochem.* **1999,** 77, 3-12.
6. Kratochwil, N. A.; Bednarski, P. J. Relationships between reduction properties and cancer cell growth inhibitory activities of cis-dichloro- and cis-diiodo-Pt(IV)-ethylenediamines. *Arch. Pharm.* **1999,** 332, 279-285.
7. Hall, M. D.; Hambley, T. W. Platinum(IV) antitumour compounds: their bioinorganic chemistry. *Coord. Chem. Rev.* **2002,** 232, 49-67.
8. Zhang, J. Z.; Wexselblatt, E.; Hambley, T. W.; Gibson, D. Pt(IV) analogs of oxaliplatin that do not follow the expected correlation between electrochemical reduction potential and rate of reduction by ascorbate. *Chem. Commun.* **2012,** 48, 847-849.
9. Davies, N. M.; Anderson, K. E. Clinical pharmacokinetics of diclofenac. Therapeutic insights and pitfalls. *Clin. Pharmacokinet.* **1997,** 33, 184-213.
10. Krause-Heuer, A. M.; Price, W. S.; Aldrich-Wright, J. R. Spectroscopic investigations on the interactions of potent platinum(II) anticancer agents with bovine serum albumin. *J. Chem. Biol.* **2012,** 5, 105-113.
11. Garbutcheon-Singh, K. B.; Myers, S.; Harper, B. W. J.; Ng, N. S.; Dong, Q.; Xie, C.; Aldrich-Wright, J. R. The effects of 56MESS on mitochondrial and cytoskeletal proteins and the cell cycle in MDCK cells. *Metallomics* **2013,** 5, 1061-1067.
